# Secretome of brain microvascular endothelial cells promotes endothelial barrier tightness and protects against hypoxia-induced vascular leakage

**DOI:** 10.1186/s10020-024-00897-6

**Published:** 2024-08-26

**Authors:** Rodrigo Azevedo Loiola, Johan Hachani, Sophie Duban-Deweer, Emmanuel Sevin, Paulina Bugno, Agnieszka Kowalska, Eleonora Rizzi, Fumitaka Shimizu, Takashi Kanda, Caroline Mysiorek, Maciej Mazurek, Fabien Gosselet

**Affiliations:** 1 UR 2465, Laboratory of the Blood-Brain Barrier (LBHE), Sciences Faculty Jean Perrin, Artois University, 62300 Lens, France; 2 Pure Biologics S.A., Duńska 11, 54-427 Wroclaw, Poland; 3 Department of Neurology and Clinical Neuroscience, Graduate School of Medicine, Yamaguchi University, Ube, Japan

**Keywords:** Brain microvascular endothelial cells, Secretome, Blood–brain barrier, Stroke, Angiogenesis, Cell therapy, Cardiovascular disease, Regenerative medicine

## Abstract

Cell-based therapeutic strategies have been proposed as an alternative for brain and blood vessels repair after stroke, but their clinical application is hampered by potential adverse effects. We therefore tested the hypothesis that secretome of these cells might be used instead to still focus on cell-based therapeutic strategies. We therefore characterized the composition and the effect of the secretome of brain microvascular endothelial cells (BMECs) on primary in vitro human models of angiogenesis and vascular barrier. Two different secretome batches produced in high scale (scHSP) were analysed by mass spectrometry. Human primary CD34^+^-derived endothelial cells (CD34^+^-ECs) were used as well as in vitro models of EC monolayer (CMECs) and blood–brain barrier (BBB). Cells were also exposed to oxygen–glucose deprivation (OGD) conditions and treated with scHSP during reoxygenation. Protein yield and composition of scHSP batches showed good reproducibility. scHSP increased CD34^+^-EC proliferation, tubulogenesis, and migration. Proteomic analysis of scHSP revealed the presence of growth factors and proteins modulating cell metabolism and inflammatory pathways. scHSP improved the integrity of CMECs, and upregulated the expression of junctional proteins. Such effects were mediated through the activation of the interferon pathway and downregulation of Wnt signalling. Furthermore, OGD altered the permeability of both CMECs and BBB, while scHSP prevented the OGD-induced vascular leakage in both models. These effects were mediated through upregulation of junctional proteins and regulation of MAPK/VEGFR2. Finally, our results highlight the possibility of using secretome from BMECs as a therapeutic alternative to promote brain angiogenesis and to protect from ischemia-induced vascular leakage.

**Supplementary Information:**

The online version contains supplementary material available at 10.1186/s10020-024-00897-6.

## Background

Ischemic stroke is among the leading causes of mortality and disability worldwide (Prabhakaran et al. 2015). In the past decades, recanalization therapy has dramatically reduced the mortality and functional disabilities (Prabhakaran et al. 2015). However, there are no successful therapies targeting brain repair or vascular remodelling after stroke. Cerebral ischemia results in irreversible damage not only at the neuronal level but also in the brain microvasculature, namely the blood–brain barrier (BBB) (Abdullahi et al. 2018). Brain microvascular endothelial cells (BMECs) forming a monolayer in brain microvessels are a major component of the BBB, which acts as a physical barrier due to the presence of tight junctions (TJ) between adjacent endothelial cells (ECs) and the absence of fenestration and pinocytic activity of these cells (Abbott et al. 2006; Gosselet et al. 2021). Besides, the delivery of essential nutrients to the brain parenchyma is strictly regulated by specific enzymes, receptors, and efflux pumps expressed at the luminal face of the BBB ECs (Gosselet et al. 2021). BBB impairment and vascular disruption are early events following an ischemic stroke, exacerbating the brain injury and contributing to cognitive impairment (Abdullahi et al. 2018; Arai et al. 2009). In this context, developing new therapies that combine the protection of the BBB integrity and the promotion of angiogenesis could be a potential strategy to improve the functional outcome after stroke. This hypothesis is supported by a body of evidence suggesting that therapies promoting angiogenesis can stimulate neurogenesis and improve brain repair after stroke (Ergul et al. 2012).

Over the last years, cell therapy with different cell types like pericytes, stem cells from various origins or microglia has been proposed as an alternative approach to promote angiogenesis or brain repair after stroke and to decrease neuroinflammation (Wechsler et al. 2018; Hossein Geranmayeh et al. 2023). Although several pre-clinical studies have demonstrated that these cell-based therapies potentiate neurogenesis and angiogenesis in mouse models of stroke (Taguchi et al. 2004; Bai et al. 2015; Morancho et al. 2013), the clinical translation of this approach still raises safety concerns due to their potential adverse side effects and limitations depending on the injection method (Wechsler et al. 2018; Boltze et al. 2015). When injected intravenously, cells do not cross easily the BBB and are cleared by the liver and the lungs. Intra arterial injection of cells provokes microthrombi and do no improve the BBB crossing (Nistor-Cseppentö et al. 2022). Invasive methods such intracerebral and intrathecal methods might cause additional brain damages and haemorrhages (Wechsler et al. 2018).

In this context, it has been demonstrated that administration of cell-conditioned medium (aka secretome), instead of directly the cells, promotes a beneficial response in pre-clinical models of cerebral ischemia and hypoperfusion without the limitations cited above (Rosell et al. 2013; Maki et al. 2018). Despite these promising results, mainly obtained with animal cells and models, very few studies have been designed to characterize these secretomes and their effects on human cells, in particular in angiogenesis and barrier properties of ECs. In addition, few studies have been investigated the potential of secretomes from BMECs composing the BBB to promote angiogenesis and to decrease BBB opening in stroke. The present study was therefore designed to evaluate the effect of BMEC-secretome on in vitro models of angiogenesis and ischemia using primary human ECs. Our findings suggest that BMECs produce modulatory molecules which promote angiogenesis and vessel maturation while preventing the hypoxia-induced vascular leakage in oxygen–glucose deprivation (OGD) conditions in vitro. Altogether these data support the use of BMEC-secretome to improve microvascular repair in the human brain after stroke.

## Methods

### Reagents

EC medium (ECM) and EC growth supplement (ECGS) were purchased from Sciencell (USA); fetal bovine serum (FBS) from Gibco (France); Dulbecco’s modified Eagle medium (DMEM), Endothelial cell growth factor (EGM), vascular endothelial growth factor receptor 2 (VEGFR2) kinase inhibitor VII, AKT inhibitor VIII, U0126 monoethanolate, AZ6102, and fibroblast growth factor (FGF) receptor tyrosine kinase inhibitor were purchased from Sigma-Aldrich (France).

### BMEC-secretome production

#### BMEC culture

For this study, the human cortical microvessel endothelial cells (hCMEC/D3) cell line has been selected as brain microvascular endothelial cells origin. These cells were derived from human temporal lobe microvessels isolated from tissue excised during surgery for control of epilepsy, subsequently immortalized and deeply characterized (Weksler et al. 2005). These cells retained a large part of the BBB characteristics (receptor, efflux pumps, enzymes) (Uchida et al. 2011), but show low expression of tight junction proteins and then a high permeability (Helms et al. 2016). For this reason, hCMEC/D3 cell line was used to produce the secretomes but was replaced by another relevant human BBB model for permeability studies and functional tests (see below).

All flasks were previously pre-coated (37 °C, 45 min) with human fibronectin (hFN) (1:100) diluted in H_2_O and then seeded with hCMEC/D3 in EGM-2 in a 5% CO2 incubator at 37 °C. After 3 days in culture, non-adherent cells were discarded and the medium was changed every other day. Immuno-phenotyping of hCMEC/D3 was confirmed by von Willebrand factor (vWF—Dako), Ulex Europaeus Agglutinin-1 (UEA-1—Sigma-Aldrich), CD34 (Santa Cruz) and VEGFR2 (Santa Cruz), and Short Tandem Repeat (STR) analysis with result > 95%.

#### Low-scale production (LSP) of BMEC-secretome

The LSP was performed to obtain small quantities of BMEC-secretome as usually performed in academic laboratories for studying secretomes. Briefly, BMECs (3 × 10^6^ cells) were resuspended in 12 mL of complete EGM-2 and seeded in T75 flasks (75 cm^2^). After 72 h, cells were rinsed (PBS-CMF: 8 g/L NaCl, 0.2 g/L KCl, 0.2 g/L KH_2_PO_4_, 2.86 g/L Na_2_HPO_4_·12 H_2_O; pH 7.4) and incubated (1 h) with DMEM. Then, DMEM was discarded and cells were cultivated with 12 mL of Endothelial Basal Medium (EBM—Lonza) without the addition of any supplements. After 24 h, the conditioned medium was collected, filtered, concentrated and named super concentrated LSP secretome (scLSP). Six different vials of hCMEC/D3 cells were used to produce 6 different batches of scLSP (scLSP-1 to scLSP-6). All the results obtained with these different scLSP batches were merged to give the scLSP condition.

#### High-scale production (HSP) of BMEC-secretome

The HSP was performed in 10-layers culture flasks to provide higher content of proteins in the conditioned medium, which is desirable to consider future studies and treatments in patients. Briefly, BMECs (1 × 10^7^ cells) were resuspended in 25 mL of EGM-2 and seeded in a T175 flask (175 cm^2^). After 96 h, BMECs were trypsinized and split in three T300 flasks (300 cm^2^). For every T300 flask, 2 × 10^7^ cells were seeded in 70 mL of complete EGM-2 medium. After 72 h, BMECs were trypsinized, resuspended (2.48 × 10^8^ cells) in 1 L of EGM-2 and seeded in a CF10 flask (Nunc™ EasyFill™ Cell Factory™ Systems, culture area 6320 cm^2^). After 96 h (100% confluence), the EGM-2 medium was discarded and cells were rinsed with PBS-CMF. Next, cells were incubated in DMEM medium, which was discarded after 1 h and replaced with 1L of EBM without supplements. After 24 h, conditioned media was collected and filtered/concentrated, and named scHSP. Two different batches of this secretome were produced (scHSP-1 and scHSP-2).

#### Filtration and concentration of BMEC-secretome

BMEC-secretome obtained by LSP or HSP, and fresh protein-free EBM (control) were filtered through a 0.22 μm vacuum filter to remove cells and debris. Centricon Plus-70 filters (3 kDa Ultracel-PL membrane—Millipore) were filled with BMEC-secretome or EBM and centrifuged (3500×*g*) at 4 °C for 60 min. The super-concentrated BMEC-secretomes (scLSP and scHSP) or EBM (scEBM) were recovered by centrifuging in collection mode at 1000×*g* for 1 min. Finally, the protein content was determined by Bradford assay and frozen at − 80 °C in low protein binding tubes until uses.

### Treatments

All experiments were performed in absence of serum or other commercial growth factors. The BMEC-secretomes (5 μg/mL of either scHSP, or scLSP) or scEBM (control) were diluted in serum-free ECM containing bovine serum albumin (BSA) 0.1%. VEGF-A was used at 50 ng/mL. For the experiments using inhibitors, CD34^+^-ECs were pre-treated 30 min before adding scEBM or scHSP using the following concentrations: U0126 (MAPK inhibitor—1 μmol/L); VEGFR2 kinase inhibitor VII (10 μmol/L); AKT inhibitor VIII (1 μmol/L); AZ6102 (Wnt inhibitor—1 μmol/L); FGFR tyrosine kinase inhibitor (1 μmol/L). For experiments using TNFα, cells were pre-treated (24 h) with scHSP or scEBM, and then TNFα (10 ng/mL—Sigma-Aldrich) was added. After 24 h of TNFα treatment, a permeability assay and sample collection were performed.

### Endothelial cells

CD34^+^ cells were isolated from human umbilical cord blood and differentiated into ECs (CD34^+^-ECs), as previously described (Pedroso et al. 2011). Briefly, CD34^+^-ECs were seeded in 100 mm 1% gelatin-coated dishes with ECM containing ECGS and 5% FBS (named ECM5). After 2 days, cells were trypsinized and seeded for the experiments of angiogenesis (cell proliferation, tubulogenesis, and migration) or the preparation of CMECs and BLECs, as further described.

For the co-culture approach (in vitro BBB model), human brain pericytes (HBPs) were grown in DMEM containing 4.5 g/L d-glucose, 10% FBS, 2 mM l-glutamine, 100 Units/mL penicillin, and 100 µg/mL streptomycin, as previously described (Shimizu et al. 2011; Deligne et al. 2020). As for the hCMEC/D3 cell line, STR analysis were performed on CD34^+^-ECs and HBPs to confirm their origins and genotypes.

### Cell proliferation

CD34^+^-ECs were seeded (5 × 10^3^ cells/well) in 1% gelatin-coated 96-well plates. After 24 h, CD34^+^-ECs were serum-starved in ECM with 0.1% BSA for 6 h. Then, cells were treated (24 h) with BMEC-secretome (scLSP or scHSP) or scEBM. Cell viability was evaluated by a Resazurin assay, as previously reported (Jennings et al. 2007). ATP levels were measured with a luminescent kit (CellTiter Glo™, Promega, France) following the manufacturer’s instructions. To assess the effect of the treatment on cell proliferation/growth, CD34^+^-ECs were seeded at a low-density, and the experiments were performed during the exponential growth phase, when no cell death was observed. Cell proliferation thus represents the cell viability (relative number of living cells) of BMEC-secretome-treated CD34^+^-ECs compared to scEBM-treated cells. Cell proliferation data were represented as the relative percentage versus the scEBM (control) group.

### Wound healing assay

CD34^+^-ECs (2 × 10^5^ cells/well) were seeded in 1% gelatin-coated 24-well plates. After 24 h, CD34^+^-ECs were serum-starved in ECM with 0.1% BSA for 6 h. Then, a wound was created by scratching the cell monolayer with a 200 μL tip. Cells were rinsed with DMEM to remove cell debris and treated with BMEC-secretome (scLSP, or scHSP) or scEBM. Images of the wound were taken immediately after the scratch (time 0) and 16 h after the treatments using a phase-contrast microscope (Nikon). The wound healing of BMEC-secretome treated cells was calculated by measuring the difference between the initial (time 0) and final (time 16 h) wound area using the ImageJ software. The migration of BMEC-secretome-treated CD34^+^-ECs was calculated as the relative percentage compared to the scEBM-treated cells.

### Capillary-like tube formation assay

Angiogenesis μ-slides (IBIDI, Germany) were coated with Matrigel™ (BD Biosciences—10 μL/well) and incubated at 37 °C for 1 h, as previously described (Ma et al. 2016). After serum-starvation (ECM + 0.1% BSA) for 16 h, CD34^+^-ECs were detached and seeded (12 × 10^3^ cells/well) on the surface of polymerized Matrigel™ and treated with BMEC-secretome (scLSP, scHSP) or scEBM. After 6 h of incubation, pictures were taken using a phase-contrast microscope (Nikon) with a 5× magnification objective. The number of tubular structures was determined using the Wimasis® Image Analysis software and the tubulogenesis of BMEC-secretome treated CD34^+^-ECs was calculated as the relative percentage versus the scEBM-treated cells.

### Confluent monolayers of endothelial cells (CMECs)

Briefly, CD34^+^-ECs (8 × 10^4^ cells/insert) were seeded in Matrigel™-coated Transwell inserts (Costar Transwell inserts, pore size 0.4 μm). Filters were placed in 12-well plates containing ECM5 and after 4 days, cells were treated with scHSP or scEBM. Then, 48 h later, permeability studies and sample collection were performed.

### In vitro BBB model with brain-like endothelial cells (BLECs)

The BBB model was reproduced as previously published (Cecchelli et al. 2014). Briefly, CD34^+^-ECs (8 × 10^4^ cells/insert) were seeded into Matrigel™-coated filters (Costar Transwell inserts, pore size 0.4 μm). Then, inserts were placed in collagen-coated 12-well plates containing HBPs (5 × 10^4^ cells/well). After 5 days of co-culture, CD34^+^-ECs acquire the major BBB properties observed in vivo (Marjolein Heymans et al. 2020; Dehouck et al. 2022) and reproduce a suitable model to investigate BBB permeability and physiology (Cecchelli et al. 2014; Dehouck et al. 2022; Luo et al. 2018; Melander et al. 2023). They are therefore named as brain-like ECs (BLECs). Once differentiated, BLECs were treated with scHSP or scEBM and, 48 h later, permeability studies and sample collection were performed.

### Permeability assay

HEPES-buffered Ringer’s solution was added to empty wells in a 12-well plate (Costar). Filter inserts containing CMECs or BLECs were subsequently placed in the 12-well plate and filled with Ringer-HEPES buffer (RH) containing the fluorescent integrity marker Sodium Fluorescein (NaFlu; 10 µM; Life Technologies), which poorly crosses the BBB. Alternatively, some experiments were performed with radiolabeled sucrose-^14^C, another paracellular marker that also poorly crosses the BBB. After 1 h, filter inserts were withdrawn from the receiver compartment. Aliquots from the donor solution were taken at the beginning and the end of the experiments. The fluorescence intensity, hence, concentration of NaFlu, was determined by using a fluorescence multiwell plate reader (Synergy H1 multiplate reader, BioTek Instruments SAS, Colmar, France), using an excitation wavelength (λ) of 490 nm, and emission wavelength of 525 nm. Sucrose-^14^C radioactivity was quantified using an HIDEX 300SL scintillation counter (Sciencetec, Villebon-sur-Yvette, France). Subsequently, the permeability coefficient was calculated as previously described (Cecchelli et al. 1999). Briefly, both insert permeability (PSf, for insert only coated with Matrigel™) and permeability of inserts containing CMECs or BLECs (PSt, for insert with Matrigel™ and cells) were considered, according to the following formula: 1/PSe = 1/PSt − 1/PSf. The permeability value for the CMECs or BLECs monolayer was then divided by the surface area of the insert (1.12 cm^2^) to obtain the permeability coefficient (Pe) of each molecule (cm/min).

### Immunofluorescence

Cells were fixed with cold methanol and rinsed twice with cold PBS-CMF. Unspecific binding was blocked (30 min, RT) using a Sea Block buffer solution (Thermo Fisher Scientific). Then, cells were incubated [60 min, Room Temperature (RT)] with the primary antibodies against claudin 5 (Invitrogen, 34–1600), ZO-1 (Invitrogen, 61–7300), VE-cadherin (Abcam, Ab33168), or occludin (Invitrogen, 71–500) in PBS-CMF containing 2% (v/v) normal goat serum (PBS-NGS). After rinsing, cells were incubated (30 min, RT) with a secondary polyclonal antibody (Life Technologies, A-11034). For F actin staining, cells were fixed with 4% paraformaldehyde (PFA) and permeabilized using Triton 0.1% in PBS-CMF (10 min, RT). Then, cells were incubated (30 min, RT) with phalloidin (Bodipy—588/568—Thermo Fisher, B3475) diluted in PBS-NGS. After rising, cells were mounted using ProLong Gold antifade mountant (Thermo Fisher) containing DAPI (nuclear staining). Images were acquired using a Leica microscope (DMRD; Leica Microsystems) and processed using the ImageJ software.

### RT-qPCR

The mRNA from cells was extracted using the NucleoSpin® RNA/protein kit (Macherey–Nagel, Germany). cDNA was obtained from 250 ng of mRNA using IScript™ Reverse Transcription Supermix (BioRad, USA), following the manufacturer’s instructions. qPCR reactions (10 µL) were prepared using SsoFast™ EvaGreen® Supermix (BioRad), primers (100 nM), deionized water, and cDNA. qPCR amplification was carried out for 40 cycles with an annealing temperature of 60 °C in a CFX96 thermocycler (BioRad). Ct data were obtained using the Bio-Rad CFX Manager software. Gene expression levels of the targets (Table 1) were calculated using the 2^ΔΔCt^ method, relative to the housekeeping gene *PPIA* (Cyclophilin A).

**Table 1:** List of primers used in the study and their corresponding sequence

Target	Gene	Primer sequence	
Cyclin D1	*CCND1*	Forward:	GAAGATCGTCGCCACCTGGA
		Reverse:	CAGGCGGCTCTTTTTCACGG
APC down-regulated 1	*APCDD1*	Forward:	ACTGATGCCACCCAGAGGATG
		Reverse:	AGATGATCCGACAGGCGATGC
Axin 2	*Axin 2*	Forward:	CCTGGGGGCAGCGAGTATTA
		Reverse:	TTGGGCAAGGTACTGCCTCT
Vascular endothelial growth factor A	*VEGFA*	Forward:	AGAAGGAGGAGGGCAGAATC
		Reverse:	ACACAGGATGGCTTGAAGATG
Cyclophilin A	*PPIA*	Forward:	CTGAGGACTGGAGAGAAAGGAT
		Reverse:	GAAGTCACCACCCTGACACATA
Glucose transporter 1 (GLUT1)	*SLC2A1*	Forward:	CTTCTCCAACTGGACCTCAAAT
		Reverse:	AGGAGCACAGTGAAGATGATGA
Hypoxia-induced factor 1α (HIF1α)	*HIF1A*	Forward:	GGATCAGACACCTAGTCCTT
		Reverse:	ATCCATTGGGATATAGGGAG

### Western Blotting (WB)

Cells were collected with RIPA lysis buffer containing protease and phosphatase inhibitors (Sigma-Aldrich). Cell lysates (10–20 μg) were prepared, placed on sodium dodecyl sulfate–polyacrylamide gel electrophoresis (SDS-PAGE) and then transferred to nitrocellulose membranes (GE Healthcare, Germany). Nonspecific binding was blocked using tris-buffered saline containing 0.1% Tween 20 (TBS-T) with 5% of skimmed milk (1 h, RT). Membranes were incubated (4 °C, overnight) with primary antibodies (Table 2), washed extensively, and then incubated (1 h, RT) with a horseradish peroxidase-conjugated secondary antibody (Dako/Agilent Technologies). After rinsing, membranes were developed with a chemiluminescence reagent (GE Healthcare) and images were acquired using the WB Imaging System Azure c600 (Azure Biosystems). The software TotalLab TL 100 1D gel Analysis was used for quantification of the relative immunoblots densities. Conditions and concentrations of each antibody shown in Table 2 were previously optimized, as demonstrated in Additional file 1: Figure S1. Western blot images shown in this study were cropped, but original results obtained for each experiment performed are provided in Additional file 2: Figure S2, Additional file 3: Figure S3, Additional file 4: Figure S4, Additional file 5: Figure S5, Additional file 6: Figure S6, Additional file 7: Figure S7, Additional file 8: Figure S8, Additional file 9: Figure S9, Additional file 10: Figure S10, Additional file 11: Figure S11. More pictures and data can be provided upon request.

**Table 2:** List of antibodies used in the study

Target	Reference	Provider
COX-2	AF4198	R&D systems
Phospho-VEGFR2	AF1766	R&D systems
Phospho-AKT	MAB887	R&D systems
pan-VEGFR2	sc6251	SantaCruz
Non-phospho(active)-β catenin	19,807	CellSignalling
Phospho-ERK1/2	9106	CellSignalling
pan-ERK1/2	9102	CellSignalling
pan-AKT	4691	CellSignalling
pan-β catenin	Ab6302	Abcam
BCRP	Ab3380	Abcam
ICAM-1	Ab53013	Abcam
VCAM-1	Ab98954	Abcam
Tricellulin	Ab253067	Abcam
ABCA1	Ab18180	Abcam
β-actin	A5541	Sigma Aldrich
P-gp	C219	Genetex
VE-cadherin	Ab33168	Abcam
ZO-1	Ab216880	Abcam
Claudin 5	Ab15106	Abcam
Occludin	Ab31721	Abcam

### In vitro oxygen–glucose deprivation (OGD) assay

The in vitro OGD model was designed to simulate the in vivo stroke conditions, with a shortage of oxygen and nutrients (1% O_2_, 5% CO_2,_ 94% N_2_ glucose/serum free medium with supplement), which were achieved by using a hypoxic chamber (Hypoxystation H35, Whitley H35). In parallel, experiments performed under normoxic conditions (5% CO_2_/95% air and serum free medium with supplement and 1 g/L glucose) were used as controls. All media and solutions used for the OGD conditions were previously equilibrated in the hypoxic chamber. Then, inserts were submitted to OGD or normoxic conditions for 6 h and permeability assays or sample collection were performed. In another set of inserts, cells exposed to OGD or normoxic conditions were treated with scHSP or scEBM and submitted to a reoxygenation phase (24 h), which consists in returning the cells to physiological conditions (5% CO_2_/95% air using medium containing 1 g/L glucose, 5% serum and supplement) thereby mimicking the in vivo reperfusion phase. After reoxygenation, permeability assay and sample collection were performed.

### scHSP protein profile

The protein content was analysed in two independent batches of scHSP using the Proteome Profiler Human Angiogenesis Array kit (R&D Systems, USA), which can detect the expression of 55 angiogenesis-related proteins. Briefly, scHSP (150 μg of total protein) was mixed with the biotinylated detection antibodies provided in the kit and incubated (4 °C, overnight) in a nitrocellulose membrane containing the capture antibodies. After incubation, the membranes were washed and the Streptavidin-HRP and chemiluminescent detection reagents were applied. The spot signal was detected with the Luminescent Imaging System Azure c600 (Azure Biosystem) and quantification of the relative densities of the bands was performed using the TotalLab TL 100 1D gel Analysis software. scEBM was used as a negative control. Results are expressed as the percentage of the signal relative to the reference spot (loading control).

### Proteomic analysis of scHSP

#### Preparation of the samples

Proteins were extracted from 2 independent batches of scHSP (~ 100 µg each) in a 1.5 M Tris–HCl buffer (pH 8.5) containing 7 M guanidine hydrochloride (GuHCl), 20 mM ethylenediaminetetraacetate (EDTA), and 0.5 M dithiothreitol (DTT), and incubated for 1 h at 60 °C. The sulfhydryl groups of the proteins were carbamidomethylated with iodoacetamide used in a 2.5-fold excess (w/w) to DTT in the dark (20 min, RT). The suspension was centrifuged at 11.000×*g* for 15 min at 4 °C and the supernatant was collected. Protein concentration was measured using the Quick start Bradford dye reagent (Biorad, Hercules, USA) with BSA as a standard protein. For each sample, 100 μg of protein were precipitated in 80% acetone overnight at − 20 °C. After 15 min of centrifugation at 11.000 g, the pellet was enzymatically digested overnight using a sequencing grade modified trypsin (Promega, Madison, USA) with an enzyme/substrate ratio of 1:50 at 37 °C in 25 mM Ammonium Bicarbonate (NH_4_HCO_3_). The reaction was stopped by adding formic acid to a final concentration of 0.1% (v/v). Peptides were extracted using the HyperSep SpinTip Microscale C18 (Thermofisher Scientific, USA) and the peptides concentration was estimated by using the Quantitative Colorimetric Peptide Assay (Thermofisher Scientific, USA).

#### Data-dependent acquisition by mass spectrometry (DDA-MS)

DDA consists in a proteomic approach in which digested peptides are analysed by LC-MSMS. Peptide signals that raised in a full-scan mass spectrum with predefined MS parameters were selected for fragmentation and then their products (MS/MS) mass spectra were matched to spectra in a protein database (UniProtKB). Briefly, two µg of peptides from each sample were injected using an Eksigent nano-LC 2D HPLC system connected to a quadrupole time-of-flight Triple TOF 5600+ mass spectrometer (Sciex, Redwood City, USA). Peptides are separated on a hydrophobic stationary phase (ChromXP C18, 3 μm 120 A, nanoLC column, 3C18-CL, 75 μm × 15 cm, Sciex, USA) and eluted at a flow rate of 300 nL/min with a gradient of increasing organic solvent concentration, for solvent B: 2 to 8% solvent B in A (from 0 to 5 min), 8 to 35% B (5 to 90 min), 35 to 40% B (90 to 100 min), 40 to 90% B (100 to 102 min), 90% B (102 to 107 min), 90 to 2% B (107 to 109 min) and finally 2% solvent B in A (109 to 140 min), with a total runtime of 140 min including a mobile phase equilibration. The following solvents were added in the mobile phase A: 2% acetonitrile/98% of 0.1% formic acid (v/v) in water; and in the mobile phase B: 98% acetonitrile/2% of 0.1% formic acid (v/v) in water. The eluted peptides were directly analysed for MS data acquisition in a hybrid quadrupole-TOF 5600+ System fitted with a Nanospray III source. Ionization was obtained with an ion spray voltage of 2.2 kV, curtain gas set at 25 psi, and ion source gas at 3 psi, using positive ion mode. DDA-MS survey scans were acquired at 250 ms from 400 to 1500 m/z and MS/MS scan from 100 to 2000 m/z (100 ms accumulation time, 50 mDa mass tolerance, rolling collision energy).

Peptide and protein identifications were performed using the Protein Pilot software (Version 5.0.2, Sciex) with an UniProtKB concatenated target-reverse decoy database (November 2018), specifying iodoacetamide and methionine oxidation as variable modifications and two trypsin miscleavages. The false discovery rate (FDR) was used to generate a spectral library and was set to 1% for both peptides and proteins.

### TempO-Seq analysis

Targeted transcriptome quantification assay (TempO-Seq, BioSpyder) was performed in CMECs (n = 3 per group) treated with scHSP (5 μg/mL) or scEBM (control). Cells were rinsed with sterile PBS-CMF, lysed using a TempO-Seq lysis buffer, and stored at − 80 °C before shipment to BioClavis (Glasgow, UK), where the TempO-Seq assay was performed. The TempO-Seq analysis was performed as previously described (Wellens et al. 2021). Briefly, the toxicity pathway analysis was performed using a list of genes annotated to different stress response pathways (3565 probe-set representing 3257 genes). The FASTQ file from each sample was aligned against the TempO-Seq transcriptome using the Bowtie aligner, generating a table of counts per gene and sample, which was further analysed using the R software. The differential expression analysis was performed by comparing the scHSP-treated samples with their suitable control (scEBM). Genes were considered significantly differentially expressed when the Benjamin Hochberg adjusted P value was < 0.05.

### Statistical analysis

All statistics were analysed using GraphPad Prism® software version 9.0. The normality of continuous variables was assessed using the Shapiro–Wilk test (n < 30) or Kolmogorov–Smirnov test (n > 30). For variables that were not normally distributed, Mann–Whitney or Kruskal–Wallis tests were performed and the values were expressed as median (interquartile range, 25–75). Results are represented with box plots with min, first quartile, median, third quartile and max. *p < 0.05, **p < 0.01, ***p < 0.001.

For variables that were normally distributed, student’s t-test or one-way ANOVA were performed and the values were expressed as mean ± standard deviation (SD). The threshold for statistical significance was set as p < 0.05 (*), with *p < 0.05, **p < 0.01, ***p < 0.001.

## Results

### BMEC-secretome promotes angiogenesis of CD34^+^-ECs

**Fig. 1:**
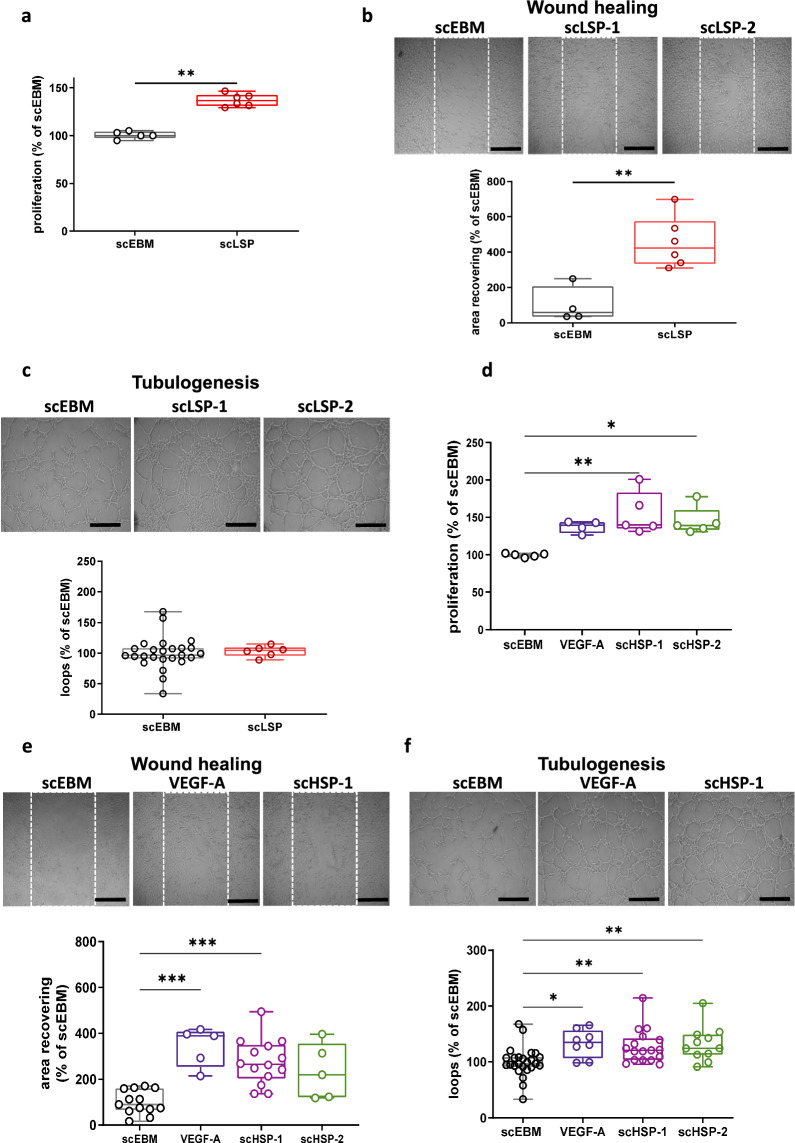
BMEC-secretome promotes in vitro angiogenesis. BMEC-secretomes under low scale production conditions (scLSP) promote CD34^+^-ECs proliferation (**a**), migration (**b**), but not tubulogenesis (**c**). Results were obtained with six different batches of scLSP (scLSP-1 to scLSP-6). Representative pictures are from scLSP-1 and scLSP-2. As indicated in the material and methods section, Wilcoxon–Mann–Whitney test has been used for **a**–**c**. Further, BMEC-secretome produced at high-scale conditions (scHSP) increased CD34^+^-ECs proliferation (**d**), migration (**e**), and tubulogenesis (**f**). Besides, two scHSP batches were compared and showed similar effects (scHSP-1, batch 1; sHSP-2, batch 2). The effects of scHSP were similar to those observed with VEGF-A (50 ng/mL). Kruskal–Wallis test has been used for **d**–**f**. Scale bar: 200 μm

To our knowledge, there is no study that investigated the ability of hCMEC/D3 secretome to promote angiogenesis of primary human ECs. Therefore, we initially compared the angiogenic properties of BMEC-secretomes from six distinct batches using LSP conditions on CD34^+^-ECs, using the well-established endothelial tube formation assay (EFTA) that is based on in vitro capillary-like growth of endothelial cells (Montesano et al. 1983; Carpentier et al. 2020). Our results suggest that these batches of scLSP promoted effect on CD34^+^-ECs proliferation (scEBM: 100.0% [97.5–100.4] and scLSP: 136.7% [131.0–142.7]) (Fig. 1a), migration (scEBM: 57.6% [35.4–207.0] and scLSP: 423.5% [332.8–575.9]) (Fig. 1b) compared to scEBM, whereas no significant effect on tubulogenesis was observed (Fig. 1c).

Then, we developed a method to obtain BMEC-secretome under HSP conditions (scHSP) which provided a higher yield of protein content (168-fold increase) in comparison to LSP conditions (LSP: 207 ± 13.6 μg vs HSP: 34.9 ± 1.6 mg). Two batches of scHSP were produced, scHSP-1 and scHSP-2. As a positive control, we included the use of Vascular endothelial growth factor (VEGF) that is well known to promote endothelial proliferation and angiogenesis processes (Kliche and Waltenberger 2001). These 2 batches promoted a consistent increase on CD34^+^-EC proliferation (scEBM: 106.8% [97.8–115.8], scHSP-1: 139.6% [134.8–183.4] and scHSP-2: 139.1% [133.2–159.9]) (Fig. 1d), migration (scEBM: 89.9% [65.6–161.2], scHSP-1: 264.3% [203.4–349.6] and scHSP-2: 219.7% [121.2–354.9]) (Fig. 1e), and tubulogenesis (scEBM: 69.7% [91.0–108.3], scHSP-1: 120.7% [103.3–142.4] and scHSP-2: 124.3% [112.8–148.8]) (Fig. 1f), which was similar to the results observed with VEGF-A (proliferation, 139.6% [128.8–143.6], migration 388.7% [252.9–406.7] and tubulogenesis 134.8% [106.4–156.3]). Besides, we observed similar effects when comparing the two different scHSP batches. The positive effect of scHSP-1 and scHSP-2 on CD34^+^-ECs viability was further confirmed by measuring the ATP content, a complementary assay for measuring cell viability (Additional file 12: Figure S12).

### scHSP characterization

**Fig. 2:**
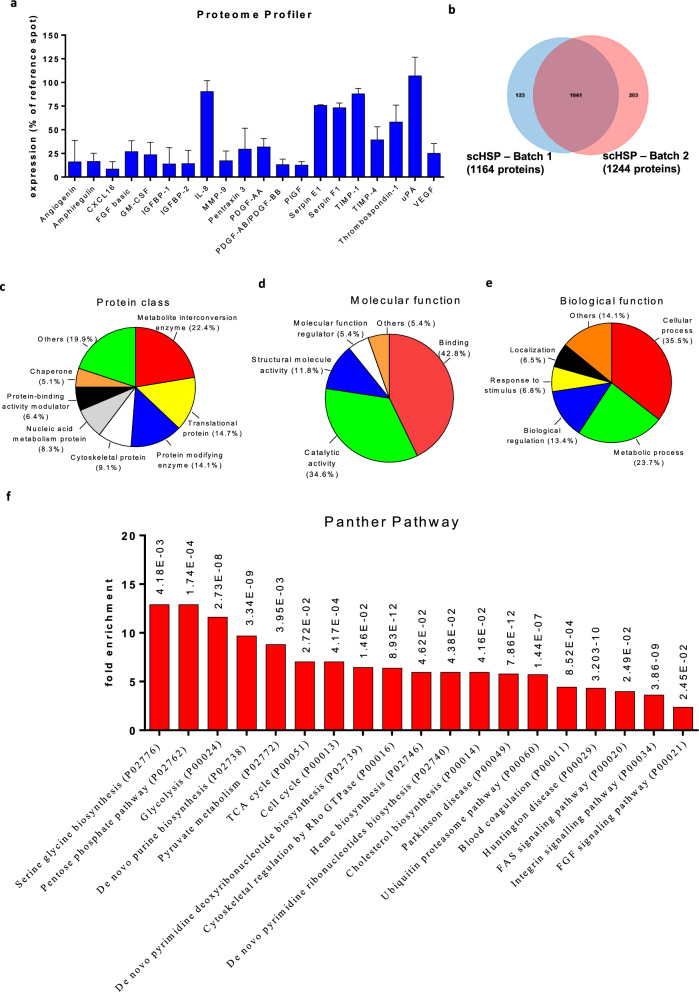
Identification of proteins in scHSP. Detection of pro-angiogenesis factors in two batches of scHSP was performed by Angiogenesis Proteome Profiler (**a**). Two different batches of scHSP were analysed by mass spectrometry (DDA-MS method). Over 1200 proteins were identified and the two batches of scHSP shared 1041 proteins (**b**). The set of proteins shared between two batches of scHSP (1041) were converted in gene identifiers and then submitted to an enrichment analysis using the GeneOntology/Panther software. The proteins were classified according to protein class (**c**), molecular (**d**), or biological function (**e**). Among the Panther pathways that were over-represented in the enrichment analysis of scHSP samples, several were related to cell metabolism (**f**)

A multiplex antibody assay was performed for the detection of angiogenesis-related proteins in the two scHSP batches. Among the investigated targets (Veldhuis et al. 2003), we detected the presence of 20 proteins involved in angiogenesis (Fig. 2a), including growth factors such as VEGF-A, FGF, angiogenin, and platelet-derived growth factor (PDGF). Moreover, we detected the presence of molecules involved in inflammatory responses (interleukin 8—IL8; CXCL16; Serpin E1) and modulators of extracellular matrix degradation (metalloproteinase 9—MMP9; tissue inhibitor of metalloproteinase 1 and 4—TIMP1 and TIMP4; urokinase-type plasminogen activator—uPA). Interestingly, we detected consistent levels of Serpin F1 and thrombospondin, which are well-known to play an anti-angiogenic activity (Belkacemi and Zhang 2016; Lawler and Lawler 2012). Next, a MS analysis of two scHSP batches revealed 1041 common proteins (Fig. 2b). By using the Panther software (Protein Analysis THrough Evolutionary Relationships, version 16.0), such proteins were converted in gene identifiers and were then classified according to their protein class (Fig. 2c), molecular function (Fig. 2d), and biological process (Fig. 2e). Besides, a Gene Ontology (GO) enrichment analysis revealed an overrepresentation (fold enrichment) of pathways involved in cell metabolism, such as serine glycine biosynthesis, pentose phosphate pathway, glycolysis, pyruvate metabolism, and TCA cycle (Fig. 2f and Supplementary Table 1).

### scHSP-induced angiogenesis is mediated through MAPK kinase and receptor tyrosine kinase (RTK) activation

**Fig. 3:**
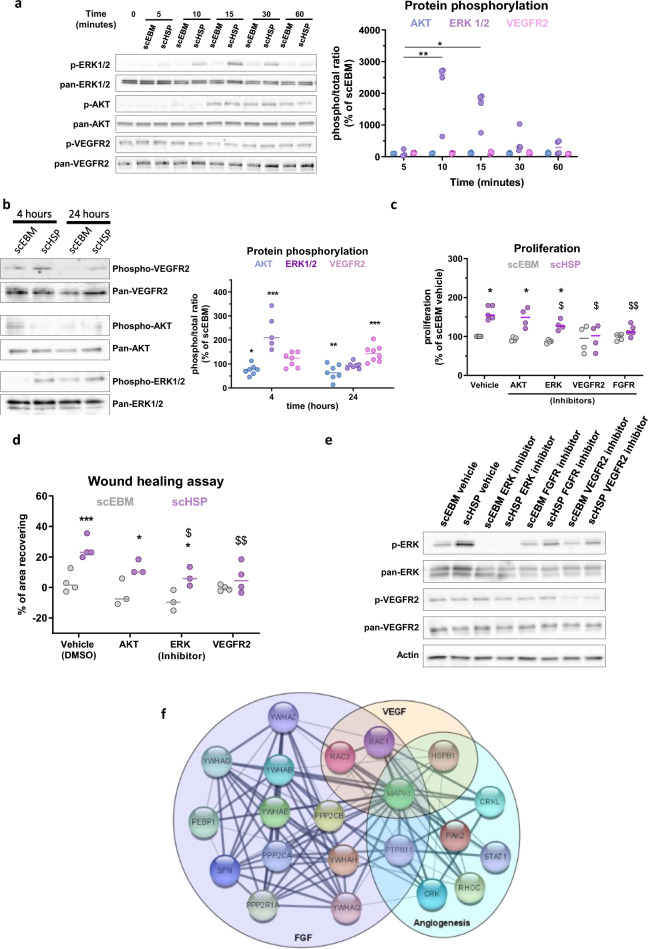
scHSP promotes in vitro angiogenesis mediated by MAPK and VEGFR2 activation. scHSP induces a rapid and consistent increase in ERK1/2 phosphorylation, whereas it does not affect AKT or VEGFR2 activation (**a**). ERK1/2 activation remains until 4 h after treatment, while VEGFR2 phosphorylation is upregulated after 24 h of treatment (**b**). Pre-incubation with U0126 (MEK/ERK inhibitor—1 μmol/L), VEGFR2 kinase inhibitor VII (10 μmol/L) and FGFR tyrosine kinase inhibitor (1 μmol/L) reduced the scHSP-induced proliferation (**c**) while VEGFR2 and FGFR inhibition reduced the scHSP-induced migration (**d**). Western blot detection of ERK1/2 and VEGFR2 phosphorylation on cells treated with either vehicle (DMSO), MEK/ERK1/2, or VEGFR2, or FGFR inhibitors (**e**). Proteins classified as involved in angiogenesis (Hossein Geranmayeh et al. 2023), VEGF-A (Gosselet et al. 2021), and FGF (Maki et al. 2018) were grouped, then converted in gene identifiers and an interaction network was prepared using Cytoscape software (version 3.8.2, released 2020.10.24). Such pathways share in common the protein MAPK1 (ERK2) (**f**). Data represents median with individual data (n = 3–7), *p < 0.05, **p < 0.01, ***p < 0.001, vs scEBM for all graphs; and ^$^p < 0.05, ^$$^p < 0.01, vs scHSP vehicle for **c** and **d**

To further investigate the underlying mechanisms of the observed scHSP-induced effects, we evaluated the activation of MAPK, VEGFR2, and PI3K/AKT signalling pathways, key modulators of angiogenesis. scHSP induced a strong and consistent ERK1/2 phosphorylation (scHSP, 10 min: 2494.0% [638.3–2718.3]), whereas it did not affect either AKT or VEGFR2 phosphorylation (Fig. 3a). Further, we observed that ERK1/2 activation lasts for at least 4 h, getting normalized after 24 h of treatment (Fig. 3b). Interestingly, AKT activation is reduced at 4 and 24 h after scHSP treatment, while VEGFR2 phosphorylation is increased after 24 h of treatment (Fig. 3b). Inhibition of ERK pathway (using U0126), VEGFR2 (using VEGFR kinase inhibitor VII), and FGF receptor (using FGFR tyrosine kinase inhibitor) attenuated the scHSP-induced increase on CD34^+^-EC proliferation (Fig. 3c). Additionally, VEGFR2 and FGFR inhibition reduced the scHSP-induced migration (Fig. 3d). The efficiency of ERK1/2 and VEGFR2 inhibition was confirmed by WB (Fig. 3e). STRING analysis database (version 11.0, released 2019.01.19) was used to classify the scHSP proteins as involved with “angiogenesis” (8 proteins), “VEGF-A pathway” (4 proteins), and “FGF pathway” (15 proteins). Among them, most were adapter proteins (14-3-3 superfamily and CRK), GTPases (RHOC, RAC1, and RAC3), or serine/threonine-protein phosphatases (PPP2R1A, PP2CA, and PPP2CB). By creating an interaction map among these proteins, we observed that MAPK1 (ERK2) was central in the network hub (Fig. 3f).

### scHSP promotes vascular tightness

**Fig. 4:**
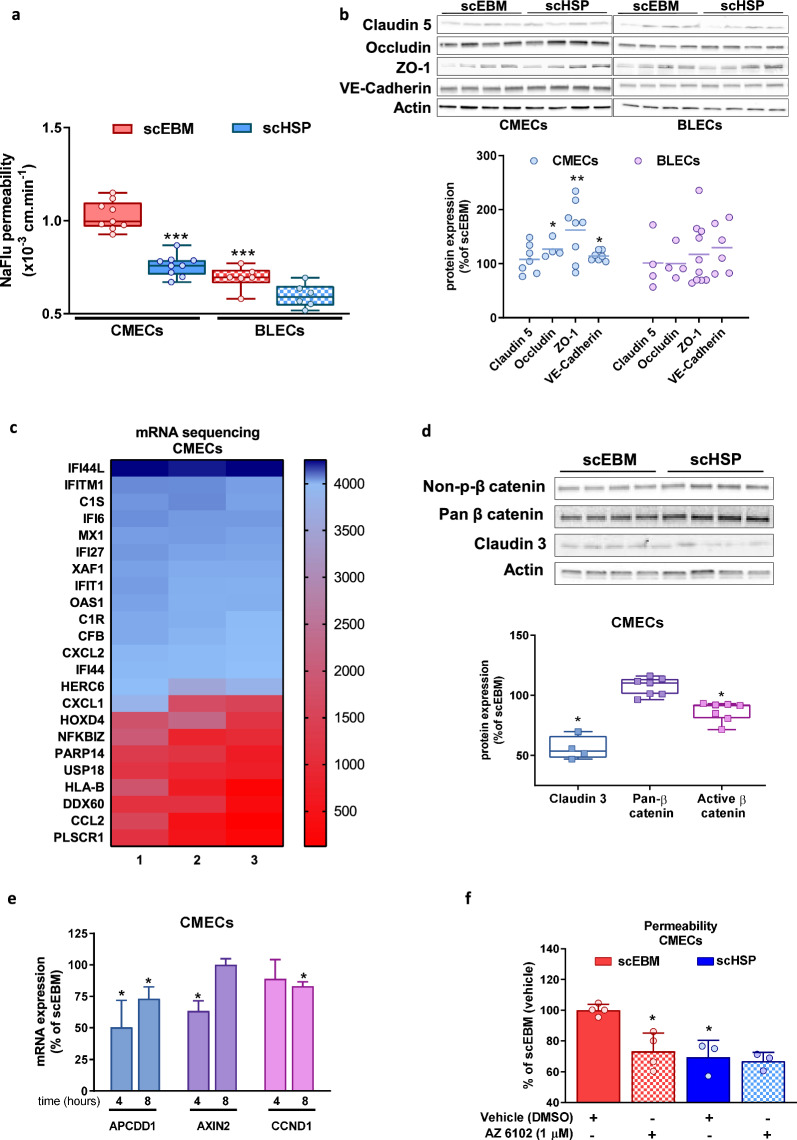
scHSP promotes endothelial barrier tightness. scHSP reduced the permeability of CMECs, while it did not affect BLECs (**a**). One way ANOVA was performed with post hoc Tukey’s multiple comparisons test, versus scEBM CMECs. scHSP up-regulated expression of junctional proteins on CMECs (**b**). Results represent Scatter dot plot. Individual data and median. Heat map representing 23 genes up-regulated by scHSP on CMECs, most of them associated with the IFN pathway (**c**). scHSP reduced the content of active β-catenin and claudin 3 (**d**) and downregulated mRNA expression of Wnt pathway targets APCDD1, Axin2, and CCND1 on CMECs (**e**). Co-incubation with Wnt inhibitor (AZ6102, 1 μmol/L) decreased barrier permeability of cells treated with scEBM (**f**)

To understand the effect of scHSP on vascular permeability, CMECs were used as a model of newly-formed vessels, which presents an elevated permeability in comparison to fully matured BBB microvessels as we demonstrated previously (Cecchelli et al. 2014). In parallel, BLECs were used as an in vitro BBB model, characterized by the presence of TJ and an extremely low permeability (Cecchelli et al. 2014). Indeed, the crossing of sucrose ^14^C, a classical paracellular marker for permeability studies, showed a 2.45-fold increase through CMECs when compared to BLECs (Additional file 13: Figure S13a). Similar results were observed using the fluorescent marker Sodium fluorescein (NaFlu), although the difference between CMECs and BLECs was more discrete (+ 50%) (Fig. 4a). However, given the concerns associated with the manipulation and disposal of radioactive tracers, further experiments were performed with NaFlu. At such conditions, VEGF-A (50 ng/mL) promoted a consistent increase in the permeability of both CMECs (2.7-fold increase) and BLECs (2.3-fold increase) (Additional file 13: Figure S13b). scHSP treatment decreased CMECs permeability (scEBM: median is 1.03 × 10^–3^ cm/min vs scHSP: 0.76 × 10^–3^ cm/min), while it did not significantly affect BLECs. WB analysis showed that scHSP increases the expression of VE-Cadherin (114.3%), ZO-1 (162.4%), and Occludin (126.8%) in CMECs (Fig. 4b), whereas it had no effect on BLECs. Immunofluorescent analysis (IF) suggested that scHSP favours the localization of TJ at the cell junctions and decreases its accumulation in the cytoplasm of CMECs (Additional file 13: Figure S13c). Besides, we observed that scHSP-treated cells presented an accumulation of F-actin fibers at the cell junctions (Additional file 14: Figure S14a, b). Furthermore, scHSP up-regulated P-glycoprotein (Pg-p) in BLECs, one of the major efflux pumps of the ABC family restricting xenobiotics entrance into the central nervous system (CNS), while it had no effect in Breast Cancer Resistant Protein (BCRP) and ABC subfamily A member 1 (ABCA1) in neither CMECs nor BLECs (Additional file 14: Figure S14c).

### scHSP upregulates the expression of genes involved in the interferon pathway

We further investigated the underlying mechanisms of scHSP effects in CMECs by performing an mRNA sequencing by TempO-seq. scHSP upregulated the expression of 23 genes (Fig. 4c). By analysing the up-regulated genes using the Panther software, we observed an overrepresentation of genes involved in interferon (IFN) pathways (Table 3 and Supplementary Table 2) according to the Reactome database. Given that previous studies have suggested that IFN response can inhibit the Wnt pathway activation (Li et al. 2012), we investigated the effect of scHSP on Wnt signalling. The levels of non-phosphorylated β-catenin, which translocates into the nucleus and transduces the Wnt pathway signalling, were reduced in scHSP-treated cells (91.5% [80.8–92.6]) (Fig. 4d) together with a downregulation of claudin 3 (53.7% [48.1–66.2]), a protein which is positively correlated with Wnt activation (Shawahna et al. 2017; Dithmer et al. 2024) (Fig. 4d). Further, we observed a reduction in the mRNA expression of Wnt pathway targets (APCDD1, Axin 2, CCND1) (Fig. 4e). Besides, Wnt pathway inhibitor (AZ6102, 1 μmol/L) did not affect scHSP-treated cells, while it caused a consistent decrease (scEBM vehicle: 100 ± 3.7% vs scEBM AZ6102: 73.2 ± 11.8%) in the permeability of scEBM-treated cells (Fig. 4f).

**Table 3:** Pathways overrepresented in CMECs treated with scHSP

Reactome pathways	Homo sapiens (n)	scHSP (n)	scHSP (expected)	Fold enrichment	*p*-value
Interferon alpha/beta signaling (R-HSA-909733)	67	9	0.07	> 100	1.25E−13
Interferon signaling (R-HSA-913531)‬‬‬‬‬‬‬‬‬‬‬‬‬‬‬‬‬‬‬‬‬‬‬‬‬‬‬‬‬‬‬‬‬‬‬‬‬‬‬‬‬‬‬‬‬‬‬‬‬‬‬‬‬‬	196	9	0.22	41.12	1.19E−09
Cytokine signaling in immune system (R-HSA-1280215)	823	12	0.92	13.06	3.34E−08
Interleukin-10 signaling (R-HSA-6783783)	45	3	0.05	59.70	4.20E−02
Antiviral mechanism by IFN-stimulated genes (R-HSA-1169410)	79	4	0.09	45.34	4.22E−03

Up-regulated genes (23 genes) in scHSP-treated CMECs were analysed by the Panther software based on the human database. Pathways (Reactome database) which were overrepresented (fold enrichment) were mainly related to IFN and inflammatory pathways. N: number of genes found in the human genome (*Homo sapiens*) or scHSP; expected: number of genes expected for a certain pathway based on the Reactome database; fold enrichment: scHSP (n)/scHSP(expected)

### scHSP protects against OGD-induced barrier disruption

Next, CMECs and BLECs were exposed to OGD mimicking the conditions observed after stroke. OGD did not affect CMECs (Additional file 15: Figure S15a), while it induced a marked increase in the permeability of BLECs (Normoxia: 0.66 [0.63–0.69] vs OGD: 1.18 [0.92–1.25] × 10^–3^ cm/min) (Additional file 15: Figure S15b). Besides, OGD up-regulated the expression of hypoxia-target genes (HIF1A, VEGF-A, and GLUT1) in both CMECs and BLECs (Additional file 15: Figure S15c). While we could not detect any marked effect of OGD on TJ expression (Additional file 14: Figure S14d), IF suggested that OGD changed the distribution of junctional proteins (Additional file 15: Figure S15e, f).

**Fig. 5:**
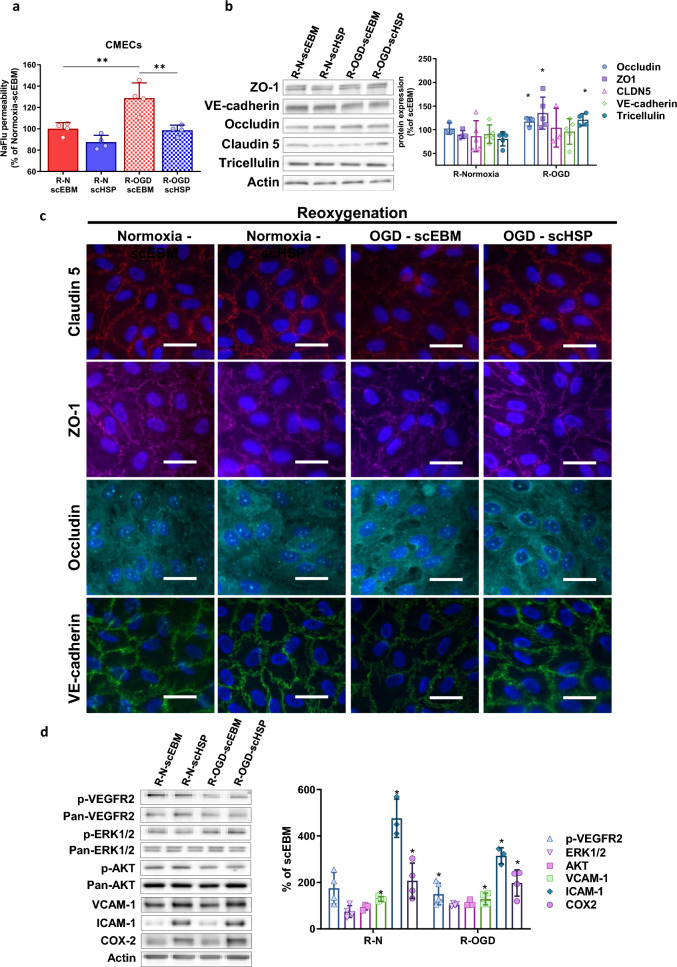
OGD-induced vascular leakage in vitro is prevented by scHSP. Treatment with scHSP during reoxygenation (24 h) abolished the OGD-induced increase of permeability on CMECs (**a**). Results represent separated bar graph with means ± SD (One way ANOVA followed by Post hoc Tukey’s multiple comparisons test versus R-OGD scEBM condition). WB analysis revealed that scHSP increased occludin, ZO-1, and tricellulin expression (**b**) and favored their localization at cell junctions (**c**). scHSP increased VEGFR2 activation, while it did not affect ERK1/2 or AKT activation (**d**). Besides, scHSP up-regulated VCAM-1, ICAM-1, and COX2 expression (**d**). As indicated in the material and method section, data represent median (interquartile range) (for **b**) or mean ± SD (for **c**). Scale bar: 10 μm

To evaluate the protective effect of scHSP under ischemic conditions, cells were treated after OGD or normoxia with scEBM or scHSP during the re-oxygenation step (R-normoxia or R-OGD). CMECs exposed to OGD conditions presented a higher permeability (scEBM R-OGD: 128.7 ± 14.3%) in comparison with the control (scEBM R-Normoxia: 100 ± 5.8%) after reoxygenation, and scHSP treatment reverted the OGD-induced barrier leakiness (scHSP R-OGD: 98.6 ± 5.0%) (Fig. 5a). WB analysis revealed that scHSP up-regulated occludin (120.3% [107.5–123.8]), ZO-1 (123.2% [107.7–168.5]), and tricellulin (120.1% [109.8–133.7]) expression (Fig. 5b). Besides, IF analysis suggested that scHSP treatment preserved the TJ distribution at the cell boundaries (Fig. 5c). Interestingly, the VEGFR2 phosphorylation was increased in scHSP-treated CMECs, with no changes detected in ERK1/2 nor AKT activation (Fig. 5d). Besides, scHSP induced the expression of VCAM-1, ICAM-1, and COX2 in CMECs during reoxygenation (Fig. 5d).

**Fig. 6:**
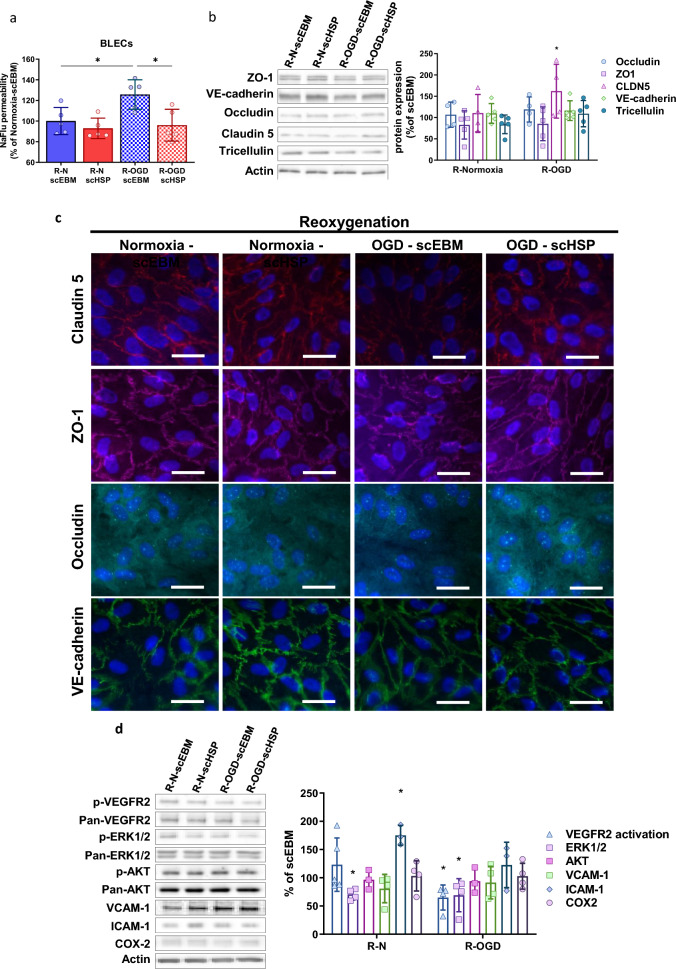
OGD-induced in vitro BBB leakage is prevented by scHSP. Treatment with scHSP during reoxygenation (24 h) abolished the OGD-induced increase of permeability on BLECs (**a**). Results show means ± SD, One way ANOVA and Post hoc Holm-Šídák’s multiple comparisons test, versus R-OGD scEBM condition. WB analysis revealed that scHSP increased claudin 5 expression (**b**) and favored its localization at the cell junctions (**c**). scHSP decreased VEGFR2 and ERK1/2 activation during reoxygenation (**d**). Moreover, scHSP increased ICAM-1 expression in normoxic conditions but had no effect on BLECs exposed to OGD (**d**). Data represent means ± SD, versus scEBM conditions. Scale bar: 10 μm

In BLECs, OGD exposure increased the permeability after reoxygenation (scEBM R-Normoxia: 100 ± 5.9% vs scEBM R-OGD: 125.8 ± 14.3%) and scHSP prevented the OGD-induced leakiness (scHSP R-OGD: 96.1 ± 15.4%) (Fig. 6a). Such effect is possibly associated with the up-regulation of claudin 5 (154.2% [106.9–224.7]) (Fig. 6b) and the restoration of TJ localization/assembly (Fig. 6c). Oppositely to the effects in CMECs, scHSP inhibited both VEGFR2 and ERK1/2 activation, while it had no effect in the expression of VCAM-1, ICAM-1, and COX2 under OGD conditions (Fig. 6d).

### scHSP prevents TNFα-induced BBB leakage

The exacerbation of TNFα production by perivascular cells plays a key role in neuroinflammation and vascular leakage after stroke (Doll et al. 2014). Since scHSP had a protective effect in the vascular barrier under hypoxic conditions, we investigated whether it was also able to prevent the TNFα-induced barrier breakdown. In CMECs, TNFα did not significantly affect the permeability (Additional file 16: Figure S16a). On another hand, TNFα induced a drastic elevation of BLECs permeability (scEBM control: 101.4% [93.3–106.0] vs scEBM TNFα 473% [375.2–1049.1]) and scHSP partially prevented BBB leakage (245.8% [202.9–688.3]) (Additional file 16: Figure S16b). In parallel, TNFα treatment downregulated occludin expression (scEBM control: 100 ± 32.5% vs scEBM TNFα: 39.2 ± 9.5%) and pre-treatment with scHSP partially inhibited (scHSP TNFα: 64.8 ± 25.7%) this effect in BLECs (Additional file 16: Figure S16c).

## Discussion

Ischemic stroke is a major cause of death and disability worldwide and the current therapeutic interventions targeting brain repair are still scarce. Over the last years, cell therapy with endothelial progenitor cells (EPCs) or different cell types like pericytes, or microglia has been pinpointed as a promising approach for the enhancement of both vascular remodelling and neurogenesis after stroke (Wechsler et al. 2018; Taguchi et al. 2004; Bai et al. 2015; Morancho et al. 2015). However, concerns associated with cell-based therapies have limited their application for clinical purposes. In this context, cell-free therapies have been considered as an interesting alternative and some studies have reported beneficial effects of EPC-secretome in pre-clinical models of stroke and hypoperfusion (Rosell et al. 2013; Maki et al. 2018). With all these considerations, we designed this study to investigate if secretome of brain microvessel endothelial cells (BMECs) that line the brain vasculature might be produced at high scale and might be used in a free-cell based therapy in stroke.

**Fig. 7:**
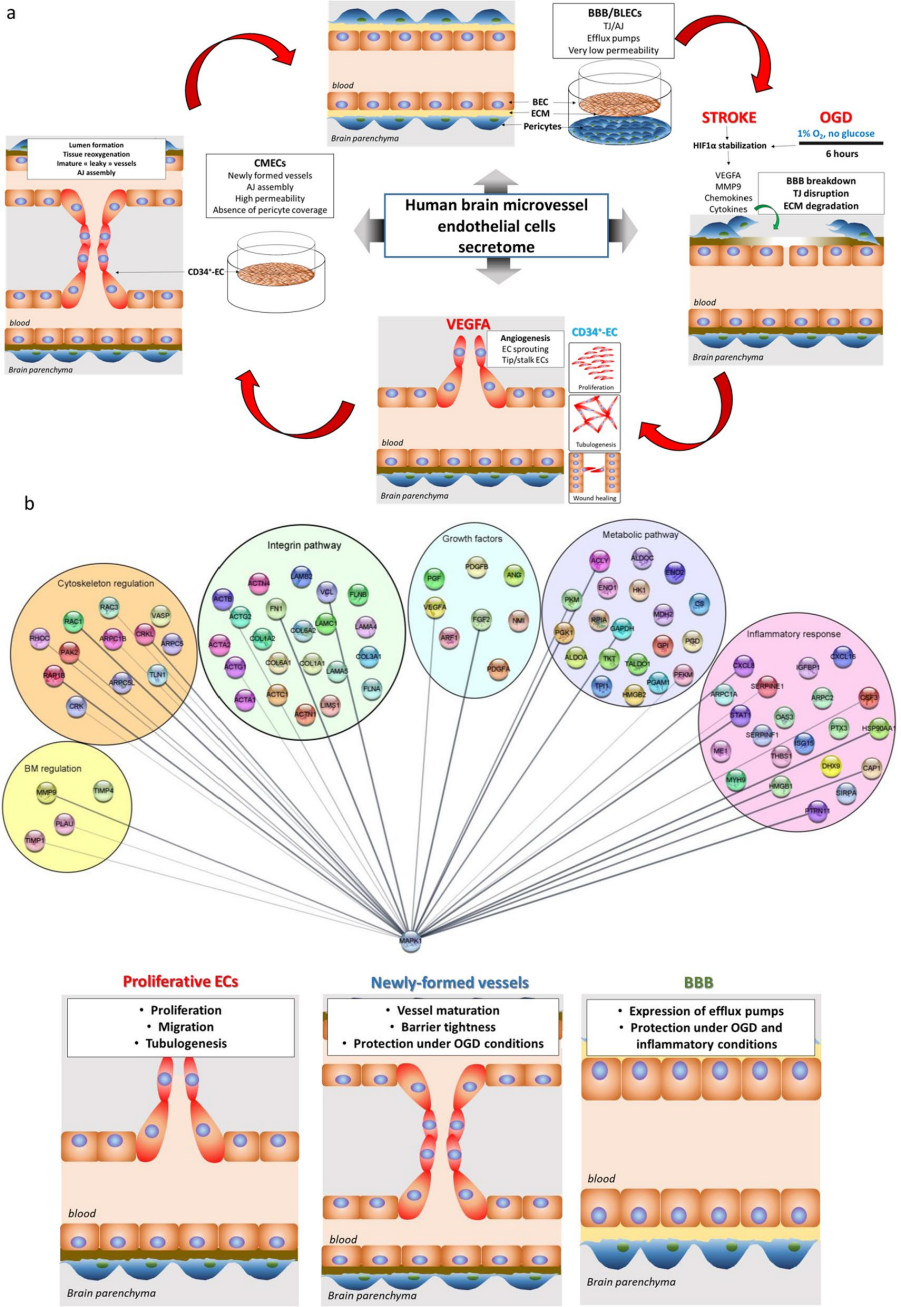
Network map of potential proteins/pathways involved in scHSP-induced effects. **a** Schematic overview of angiogenesis process and in vitro assays proposed for evaluating EPC-secretome effects. Human CD34^+^ derived cord-blood hematopoietic endothelial cells (CD34^+^-ECs) were used to study angiogenesis (proliferation, cell migration, and tubulogenesis). To investigate mechanisms involved in vascular maturation, CD34^+^-EC were seeded on the surface of Matrigel™-coated Transwell inserts and cultivated in monocultures (CMECs). Finally, to study the effect of EPC-secretome on brain capillaries, we used an in vitro BBB model which consisted in seeding CD34^+^-ECs on Matrigel™-coated Transwell inserts and co-cultivating them with human brain pericytes, which enabled CD34^+^-ECs to acquire a brain-like endothelial cell phenotype (BLECs). **b** Proteins present in scHSP were converted in gene identifiers and were then grouped according to their biological function/pathway (Metabolic pathways, Growth factors, Inflammatory response, basal membrane (BM) regulation, cytoskeletal regulation, and integrin pathway). An interaction network was prepared using the Cytoscape software (version 3.8.2, released 2020.10.24). The regulation of MAPK activity might play an essential role in the scHSP-induced angiogenesis in human primary ECs. In parallel, scHSP promotes vascular maturation of newly-formed vessels and protects the BBB integrity under ischemic and inflammatory conditions

The present study reports a reproducible method for high-scale production of BMEC-secretome (scHSP) for therapeutic purposes. Further, we investigated the effect of scHSP in primary human endothelial cells (ECs), with a particular focus on angiogenesis and the regulation of vascular permeability (Fig. 7a). Overall, our results suggested that scHSP exhibits an important angiogenic activity while lacking the negative effects of VEGF-A on vascular barrier properties. To the best of our knowledge, this is the first study evaluating the effect of BMEC-secretome produced under high-scale conditions in a human in vitro BBB model. The results from this study expand the current knowledge about the mechanisms underlying the BMEC-mediated effects on vascular cells and highlight proteins/pathways which can be targeted for the simultaneous promotion of angiogenesis and vascular protection in the brain (Fig. 7b).

The BMEC-secretome obtained under HSP conditions (scHSP) showed a higher protein concentration (168-fold increase) in comparison to traditional production method performed in laboratories (scLSP). Additionally, the composition of LSP and HSP might be slightly different, since we observed a consistent effect of high-scale produced BMEC-secretome on tubulogenesis, while the secretome produced under LSP had no effect. It is important to highlight that the standardization of HSP of BMECs-secretome consisted in several challenging steps, such as controlling cell density at seeding, change of medium, and concentration of large volumes of conditioned medium. Therefore, further studies are necessary to understand the difference on composition of the secretomes produced under low- and high-scale conditions, however we consider it is an important step towards developing cell-based therapies for stroke treatment.

Proteome profiler analysis revealed an equitable presence of key angiogenesis-related proteins in two scHSP batches, indicating low batch-to-batch variation under HSP conditions. Further, MS analysis of scHSP revealed an overrepresentation of proteins associated with the modulation of cell metabolism, which is in agreement with previous studies reporting that the beneficial effects of cell therapy are due, at least in part, to metabolism regulation of injured cells (Hayakawa et al. 2018; Islam et al. 2012; Kaza et al. 2017). Indeed, the delivery of key proteins involved in the regulation of glycolysis, pyruvate, and pentose phosphate pathways by BMECs-secretome might boost cell metabolism or rescue metabolic activity in ischemic-injured cells. Additionally, the enrichment of proteins participating in cell cycle and cytoskeleton regulation might contribute to the scHSP-induced promotion of CD34^+^-ECs proliferation, migration, and tubulogenesis, which were similar to the effects induced by VEGF-A, a key regulator of angiogenesis. The scHSP-induced angiogenesis was partially mediated through MAPK activation, whereas PI3K/AKT pathway might not play a major role on this process. The RTK activation by growth factors might be the key event leading to downstream MAPK activation since inhibition of both VEGFR2 and FGFR completely abolished scHSP-induced angiogenesis. The presence of growth factors and adapter molecules on scHSP possibly contributes to RTK/MAPK activation. Additionally, the presence of integrin ligands in scHSP might contributes to VEGFR2/ERK activation, given that integrins can increase the efficiency of RTK activation (Soldi et al. 1999) and the coupling between upstream and downstream events in the RTK-Ras-MAPK cascade (Lin et al. 1997). Besides, the crosstalk between VEGFR and integrin receptors is necessary to induce the VEGFR2-mediated angiogenesis (West et al. 2012). In summary, our results suggest that the coordinated action of growth factors, adapter molecules, and integrin ligands plays a pivotal role in scHSP-induced angiogenesis (Fig. 7b).

Llombart and colleagues previously studied secretome from hCMEC/D3 cells using the stable isotope labelling with aminoacids in cell culture (SILAC) method (Llombart et al. 2016). Secretomes were analysed in normoxia versus OGD conditions and 117 proteins were considered with good confidence regarding the identification criteria (more than 2 identified peptides and CV < 30%). We found 107 of these 117 proteins in our 2 scHSP batches. 102 are observed in both secretomes, 2 in the batch 1 and 3 in the batch 2. When normoxia secretome was compared with OGD secretome, 19 secreted proteins have been differentially produced. Five (AHNAK, ANXA1, CS, PRDX3, UAP1) were upregulated and 14 were downregulated (ANP32B, BGN, CLU, COL1A2, CFB, EFEMP1, FSTL1, GSN, IGFBP2/4/7, TIMP2, SPARC, SMOC1) (Llombart et al. 2016). These up- and downregulation were then partially confirmed in blood samples of ischemic stroke patients. Our MS approach allowed us to identify these 19 proteins in our scHSP. Interestingly, 17 of them were expressed in the 2 batches of scHSP whereas only 1 (PRDX3) was expressed in the first batch, and the last one only in the second batch (CLU). Further studies are needed to better characterize the role of each of these proteins in vascular remodelling and brain repair, and it would be very interesting to duplicate our study with the use of scHSP of OGD-treated BMECs instead of the use of untreated scHSP.

Vascular leakage following acute stroke is a major hazard that can compromise brain function as well as the therapeutic outcomes of treatments targeting brain repair. Considering that the local production of VEGF-A is critical for vascular disruption following stroke (Ma et al. 2012), we investigated the effect of scHSP on permeability using an in vitro model of newly-formed vessels (CMECs) and an already established BBB model in vitro (BLECs). As expected, VEGF-A evoked barrier leakage, while scHSP promoted a consistent decrease in CMECs permeability together with an upregulation of junctional proteins. Interestingly, scHSP upregulated P-gp expression in BLECs, suggesting that it might potentiate the transport of xenobiotics out of the brain through the BBB. Further, mRNA sequencing analysis of CMECs revealed that scHSP upregulated the expression of several IFN-related genes. This finding is consistent with the presence of several proteins related to inflammatory pathways detected in scHSP by MS analysis. Among them, STAT1 can translocate to the nucleus and activate the transcription of IFN-stimulated genes (Khodarev et al. 2012), while NMI, ISG15, and HMGB1 are IFN-induced proteins (Xu et al. 2018; Zhao et al. 2013; Rendon-Mitchell et al. 2003) (Fig. 7b). A body of evidence has highlighted a potential beneficial effect of the IFN pathway on the regulation of vessel maturation and inflammatory response. In this regard, IFN can stabilize barrier properties of in vitro BBB models (Kraus et al. 2008) and act as a modulator of cytokine networks, reducing the cytokine-induced neutrophil infiltration and attenuating BBB disruption (Kuruganti et al. 2002; Veldhuis et al. 2003). Besides, IFN-β might counteract the TJ disruption induced by the inflammatory response on brain ECs (Kuruganti et al. 2002). Such findings are in agreement with the protective effect of scHSP against TNFα-induced disruption in BLECs.

In parallel, our results suggest that scHSP-induced barrier tightness is mediated through a moderate downregulation of Wnt activation, as highlighted by a decrease in active β-catenin content. Interestingly, some studies have reported evidence showing a common modulation of the Wnt and IFN pathways in the regulation of inflammatory responses. For instance, members of the miR-34 family, which are well-known repressors of Wnt/β-catenin signalling, potentiate the induction of IFN-responsive genes and their signalling pathways (Smith et al. 2017). Another study has shown that PEGylated-IFN inhibited β-catenin translocation to the nucleus and Wnt signalling in hepatoma cell lines (Thompson et al. 2011). Overall, our results suggest that the orchestrated modulation of the Wnt pathway and IFN signalling could be an underlying mechanism involved in scHSP-induced barrier tightness under physiological conditions.

Interestingly, a previous study investigated the effect of hCMEC/D3 secretome on brain pericyte transcriptome (Kurmann et al. 2021). Authors reported upregulation of several interferon-related genes that we also identified in our experiments (IFIT1, IFI27, IFIT3, IFI6 and IFI44). Altogether, these results suggest that IFN pathway activation might be a general feature of hCMEC/D3 secretome. Besides the role of this signaling pathway in barrier tightness, we can also hypothesize that activation of IFN pathway might strengthen the immune response of the brain ECs to prevent any CNS infection by viruses.

Next, we tested the potential beneficial effect of scHSP on cells exposed to hypoxic conditions. In this regard, treatment with scHSP completely abolished the OGD-induced leakage on CMECs and BLECs. This effect was possibly mediated through the regulation of junctional proteins expression and localization, leading to vascular protection against hypoxic-induced injury. The activity of integrin ligands and modulators of the extra-cellular matrix might be involved in scHSP-mediated protection in OGD conditions since the degradation of the basal membrane involving the BBB is directly associated with ischemia-induced vascular disruption (Kwon et al. 2009). A potential activation of the IFN pathway by scHSP also might contribute to the scHSP-mediated vascular protection under ischemic conditions. Besides, the presence of small levels of growth factors in EPC-secretome might have a protective effect on injured cells, given that growth factors can promote anti-apoptotic effects (Lanfranconi et al. 2011). Indeed, scHSP increased VEGFR2 activation on CMECs exposed to OGD conditions. In parallel, scHSP upregulated the expression of adhesion molecules (VCAM-1 and ICAM-1) and COX2, known molecular targets of VEGF pathway activation (Kim et al. 2001; Akarasereenont et al. 2002). Besides, the enrichment of proteins involved in inflammatory responses might contribute to the scHSP-induced upregulation of adhesion molecules (Fig. 7b). VCAM-1 is important to promote close intercellular adhesion between ECs and pericytes and it is required for blood vessel formation (Garmy-Susini et al. 2005). Besides, COX-2 plays a pivotal role in the VEGF-induced angiogenesis (Wu et al. 2006). The scHSP-induced adhesion molecule upregulation might favour the homing of leukocytes and circulating EPCs and immune cells on injured vessels, which can further potentiate angiogenesis. On the other hand, the scHSP-induced downregulation of VEGFR2 and ERK1/2 activation in BLECs exposed to OGD conditions might have a beneficial effect, considering that hypoxia-driven VEGF-A production is the main mechanism inducing BBB leakage (Ma et al. 2012) and that MAPK activation plays a key role in vascular injury induced by ischemia (Narasimhan et al. 2009; Maddahi and Edvinsson 2010). Altogether, these results suggest that scHSP can prevent the hypoxia-induced vascular leakiness, protecting the brain from edema-related deleterious effects.

Altogether, our results suggest that the scHSP-elicited effects in proliferative and quiescent ECs were mediated through an orchestrated action of growth factors, integrin ligands, inflammatory mediators, enzymes regulating cell metabolism, and proteins regulating cytoskeleton and basement membrane composition (Fig. 7b). The regulation of the MAPK pathway seems to be a key component of scHSP-driven effects since a variety of proteins identified are directly linked to ERK1/2 activation and the scHSP-treatment leads to the activation and/or inhibition of MAPK. However, other molecules present in the BMECs secretome, such as lipids, extracellular vesicles or microRNAs, should be also considered but not addressed in this study (Saint-Pol et al. 2020; Saint-Pol and Gosselet 2019). Certainly, further studies are necessary to better understand the mechanisms of BMECs-secretome actions and identify the key molecules triggering its beneficial effects. However, our study provides valuable information to understand the underlying mechanisms of secretome use for cell-free therapies for stroke.

## Conclusions

Herein, we describe a reproducible method for a high-scale production of BMEC-secretome and report pre-clinical evidence supporting its potential benefits for regenerative medicine. In particular, our results using human primary ECs suggest that scHSP boosts angiogenesis-related processes while preserving the vascular barrier function in healthy vessels. In addition, scHSP might promote vessel maturation and restore/preserve the BBB function in ischemic or inflammatory conditions. In conclusion, our results pave the way for future clinical trials employing BMEC-secretome at high scale in order to promote brain repair after stroke.

### Supplementary Information

Supplementary Figure 1. Representative images of pilot experiments performed to test the antibodies used in the study. Black arrows show the corresponding bands for every antibody according to the molecular weight predicted by the manufacturer. (a) phospho-AKT and pan-AKT; (b) phospho-VEGFR2 and pan-VEGFR2; (c) phospho-ERK1/2 and pan-ERK 1/2; (d) non-phospho-β-catenin and pan-β-catenin; (e) ABCA1; (f) BCRP; (g) P-gp; (h) COX-2; (i) VCAM-1; (j) ICAM-1; (k) claudin 5; (l) VE-cadherin; (m) occludin; (n) ZO-1; (o) tricellulin; (p) claudin 3. For images A–D, membranes were firstly probed with phospho-antibodies and then reprobed with pan-antibodies.

Supplementary Figure 2. Images used for western blotting analysis of ERK1/2, AKT, and VEGFR2 phosphorylation in proliferative CD34^+^-EC in response to acute administration (0–60 min) of scHSP (5 μg/mL) (Fig. 3a). bFGF (basic FGF) represented in (a) was not considered for analysis.

Supplementary Figure 3. Images used for western blotting analysis of ERK1/2, AKT, and VEGFR2 phosphorylation in proliferative CD34^+^-EC in response to administration (4 or 24 h) of scHSP (5 μg/mL) (Fig. 3b).

Supplementary Figure 4. Images used for western blotting analysis of ZO-1, VE-cadherin, occludin, claudin 5, claudin 3, non-phospho-catenin, and pan-catenin in CMECs in response to administration (48 h) of scHSP (5 μg/mL) (Fig. 4b, e). scHSP-1: batch 1; scHSP-2: batch 2. Analysis of scHSP-2 were not presented in the manuscript.

Supplementary Figure 5. Images used for western blotting analysis of ZO-1, VE-cadherin, occludin, and claudin 5 in BLECs in response to administration (48 h) of scHSP (5 μg/mL) (Fig. 4b). scHSP-1: batch 1; scHSP-2: batch 2. Analysis of scHSP-2 were not presented in the manuscript.

Supplementary Figure 6. Images used for western blotting analysis claudin 5, tricellulin, VEGFR2, VCAM-1, COX-2, occludin, VE-Cadherin, ZO-1, and ERK1/2 in CMECs exposed to normoxic (N) or OGD conditions and reoxygenation (R-N-scEBM; R-N-scHSP; R-OGD-scEBM; R-OGD-scHSP) (Figs. 5 and supplementary Fig. 15).

Supplementary Figure 7. Images used for western blotting analysis of claudin 5, ZO-1, VE-cadherin, VEGFR2, VCAM, AKT, ERK1/2, and occludin in CMECs exposed to normoxic (N) or OGD conditions and reoxygenation (R-N-scEBM; R-N-scHSP; R-OGD-scEBM; R-OGD-scHSP) (Fig. 5 and supplementary Fig. 15).

Supplementary Figure 8. Images used for western blotting analysis of VEGFR2, claudin 5, ZO-1, VE-cadherin, occludin, ICAM-1, AKT, ERK1/2, tricellulin, VCAM-1, and COX2 in BLECs exposed to normoxic (N) or OGD conditions and reoxygenation (R-N-scEBM; R-N-scHSP; R-OGD-scEBM; R-OGD-scHSP) (Fig. 6 and supplementary Fig. 15).

Supplementary Figure 9. Images used for western blotting analysis of VEGFR2, occludin, VE-cadherin, ZO-1, claudin 5, ERK1/2, AKT, and ICAM-1 in BLECs exposed to normoxic (N) or OGD conditions and reoxygenation (R-N-scEBM; R-N-scHSP; R-OGD-scEBM; R-OGD-scHSP) (Fig. 6 and supplementary Fig. 15).

Supplementary Figure 10. Images used for western blotting analysis of ABCA1, BCRP, and P-gp in CMECs (a–c) and BLECs (d, e) in response to administration (48 h) of scHSP (5 μg/mL) (Supplementary Fig. 14c). scHSP-1: batch 1; scHSP-2: batch 2. Analysis of scHSP-2 were not presented in the manuscript.

Supplementary Figure 11. Images used for western blotting analysis of claudin 5, ZO-1, occludin, and VE-cadherin in BLECs treated with vehicle or TNFα (10 ng/mL) (Supplementary Fig. 16c).

Supplementary Figure 12. scHSP increases CD34^+^-ECs proliferation. scLSP (batches 1 to 6), and scHSP (batches 1 and 2) increases ATP production by CD34^+^-ECs. Data represents median (interquartile range), Kruskal–Wallis test, versus scEBM.

Supplementary Figure 13. Effect of scHSP treatment in the endothelial barrier properties. The permeability for radiolabelled-sucrose is enhanced in CMECs compared with BLECs (a). VEGF-A (50 ng/mL) induced vascular leakage in both CMECs and BLECs (b). For (a) and (b), data represents median (with interquartile range), Wilcoxon–Mann–Whitney test, versus CMECs control. Representative images of immunofluorescence performed in CMECs (c) and BLECs (d) for detection of claudin 5, ZO-1, occludin, and VE-cadherin. Scale bar: 10 μm.

Supplementary Figure 14. Effect of scHSP treatment in the endothelial barrier properties. Co-staining with phalloidin and ZO-1 suggests that scHSP promotes the accumulation of actin fibres at the cell borders (yellow arrowheads) and decreases its distribution across the cytoplasm of CMECs (a) and BLECs (b). scHSP up-regulates P-gp expression on BLECs, whereas it did not affect ABCA1 and BCRP expression (c). Data represent median (with interquartile range), Wilcoxon–Mann–Whitney test, versus scEBM. Scale bar: 10 μm.

Supplementary Figure 15. Effect of oxygen–glucose deprivation (OGD) on in vitro vascular permeability. OGD (6 h) had no effect on permeability of CMECs (a) while it induced vascular leakage in BLECs (b). OGD up-regulated the mRNA expression of target genes, such as HIF1A, VEGF-A, and Glut-1 (c). Western blot analysis did not show a marked effect of OGD on the expression of junctional proteins (d) however IF analysis suggested that OGD induces their accumulation in the cytoplasm (e). Data represent median (with interquartile range, b), or mean ± SD (a,c, and d), versus OGD. Scale bar: 10 μm.

Supplementary Figure 16. Effect of TNFα on in vitro vascular permeability. CMECs and BLECs were pre-treated (24 h) with scEBM or scHSP (5 μ/mL) and then TNFα (10 ng/mL) was administered to the cells. Whereas TNFα had no significant effect on permeability of CMECs (a), scHSP partially prevented TNFα-induced leakage in BLECs (b). WB analysis showed that scHSP partially restored the TNFα-induced downregulation of occludin in BLECs (c). Data represent median (with interquartile range, b), or mean ± SD (a, c) versus scEBM control.

Supplementary Material 17.

Supplementary Material 18.

## Data Availability

The datasets used and/or analysed during this study are available from the corresponding authors upon reasonable request.

## Abbreviations

AKT: Protein kinase B
ATP: Adenosine triphosphate
BBB: Blood–brain barrier
BCRP: Breast cancer resistant protein
BLEC: Brain like endothelial cell
BMEC: Brain microvascular endothelial cell
BSA: Bovine serum albumin
CD34+-EC: CD34+ derived cord-blood hematopoietic endothelial cell
CMEC: Confluent monolayer of endothelial cell
CNS: Central nervous system
COX-2: Cyclooxygenase 2
DDA-MS: Data dependent acquisition-mass spectrometry
DMEM: Dulbecco’s modified Eagle medium
DTT: Dithiothreitol
EBM: Endothelial basal medium
EC: Endothelial cell
ECGS: Endothelial cell growth supplement
ECM: Endothelial cell medium
EDTA: Ethylenediaminetetraacetate
EGM: Endothelial growth medium
EPC: Endothelial progenitor cell
ERK1/2: Extracellular signal-regulated kinase ½
FBS: Foetal bovine serum
FGF: Fibroblast growth factor
GuHCl: Guanidine hydrochloride
HBP: Human brain pericytes
hCMEC/D3: Human cortical microvessel endothelial cells
hFN: Human fibronectin
HSP: High-scale production
HIF1α: Hypoxia-induced factor 1α
ICAM-1: Intercellular adhesion molecule 1
IFN: Interferon
kDa: Kilodalton
LSP: Low-scale production
MAPK: Mitogen activated protein kinase
Min: Minutes
MMP9: Metalloproteinase 9
MS: Mass spectrometry
NaFlu: Sodium fluorescein
NGS: Normal goat serum
OGD: Oxygen–glucose deprivation
PBS-CMF: Phosphate buffered saline calcium magnesium free
PDGF: Platelet-derived growth factor
Pe: Permeability coefficient
PPIA: Gene name of Cyclophilin A
P-gp: P-glycoprotein
RT-qPCR: Reverse transcription quantitative polymerase chain reaction
RH: Ringer-Hepes buffer
RT: Room temperature
RTK: Receptor tyrosine kinase
scEBM: Super-concentrated endothelial basal medium
scHSP: Super-concentrated high scale production
scLSP: Super-concentrated low scale production
SLC2A1: Glucose transporter 1
TempO-Seq: Templated Oligo-Sequencing
TNFα: Tumor necrosis factor α
TIMP1/4: Tissue inhibitor of metalloproteinase 1 and 4
TJ: Tight junction
UEA-1: Ulex europaeus agglutinin-1
uPA: Urokinase-type plasminogen activator
VCAM-1: Vascular cell adhesion molecule
VEGF-A: Vascular endothelial cell growth factor A
VEGFR2: VEGF receptor 2
vWF: Von Willebrand factor
WB: Western blot
ZO-1: Zonula occludens 1

## References

[CR1] Abbott NJ, Ronnback L, Hansson E. Astrocyte-endothelial interactions at the blood–brain barrier. Nat Rev Neurosci. 2006;7(1):41–53. 16371949 10.1038/nrn1824

[CR2] Abdullahi W, Tripathi D, Ronaldson PT. Blood–brain barrier dysfunction in ischemic stroke: targeting tight junctions and transporters for vascular protection. Am J Physiol Cell Physiol. 2018;315(3):C343–56. 29949404 10.1152/ajpcell.00095.2018 PMC6171039

[CR3] Akarasereenont P, Techatraisak K, Thaworn A, Chotewuttakorn S. The expression of COX-2 in VEGF-treated endothelial cells is mediated through protein tyrosine kinase. Mediat Inflamm. 2002;11(1):17–22. 10.1080/09629350210311 PMC1781636 11926591

[CR4] Arai K, Jin G, Navaratna D, Lo EH. Brain angiogenesis in developmental and pathological processes: neurovascular injury and angiogenic recovery after stroke. FEBS J. 2009;276(17):4644–52. 19664070 10.1111/j.1742-4658.2009.07176.x PMC3712842

[CR5] Bai Y-Y, Wang L, Chang D, Zhao Z, Lu C-Q, Wang G, et al. Synergistic effects of transplanted endothelial progenitor cells and RWJ 67657 in diabetic ischemic stroke models. Stroke. 2015;46(7):1938–46. 26045601 10.1161/STROKEAHA.114.008495

[CR6] Belkacemi L, Zhang SX. Anti-tumor effects of pigment epithelium-derived factor (PEDF): implication for cancer therapy. A mini-review. J Exp Clin Cancer Res. 2016;35:4. 26746675 10.1186/s13046-015-0278-7 PMC4706649

[CR7] Boltze J, Arnold A, Walczak P, Jolkkonen J, Cui L, Wagner DC. The dark side of the force—constraints and complications of cell therapies for stroke. Front Neurol. 2015;6:155. 26257702 10.3389/fneur.2015.00155 PMC4507146

[CR8] Carpentier G, Berndt S, Ferratge S, Rasband W, Cuendet M, Uzan G, et al. Angiogenesis analyzer for ImageJ—a comparative morphometric analysis of “endothelial tube formation assay” and “fibrin bead assay.” Sci Rep. 2020;10(1):11568. 32665552 10.1038/s41598-020-67289-8 PMC7360583

[CR9] Cecchelli R, Dehouck B, Descamps L, Fenart L, Buee-Scherrer VV, Duhem C, et al. In vitro model for evaluating drug transport across the blood–brain barrier. Adv Drug Deliv Rev. 1999;36(2–3):165–78. 10837714 10.1016/S0169-409X(98)00083-0

[CR10] Cecchelli R, Aday S, Sevin E, Almeida C, Culot M, Dehouck L, et al. A stable and reproducible human blood–brain barrier model derived from hematopoietic stem cells. PLoS ONE. 2014;9(6): e99733. 24936790 10.1371/journal.pone.0099733 PMC4061029

[CR11] Dehouck MP, Tachikawa M, Hoshi Y, Omori K, Maurage CA, Strecker G, et al. Quantitative targeted absolute proteomics for better characterization of an in vitro human blood–brain barrier model derived from hematopoietic stem cells. Cells. 2022;11(24):3963. 36552728 10.3390/cells11243963 PMC9776576

[CR12] Deligne C, Hachani J, Duban-Deweer S, Meignan S, Leblond P, Carcaboso AM, et al. Development of a human in vitro blood–brain tumor barrier model of diffuse intrinsic pontine glioma to better understand the chemoresistance. Fluids Barriers CNS. 2020;17:1–15. 32487241 10.1186/s12987-020-00198-0 PMC7268424

[CR13] Dithmer S, Blasig IE, Fraser PA, Qin Z, Haseloff RF. The basic requirement of tight junction proteins in blood–brain barrier function and their role in pathologies. Int J Mol Sci. 2024;25(11):5601. 38891789 10.3390/ijms25115601 PMC11172262

[CR14] Doll DN, Barr TL, Simpkins JW. Cytokines: their role in stroke and potential use as biomarkers and therapeutic targets. Aging Dis. 2014;5(5):294–306. 25276489 10.14336/AD.2014.0500294 PMC4173796

[CR15] Ergul A, Alhusban A, Fagan SC. Angiogenesis: a harmonized target for recovery after stroke. Stroke. 2012;43(8):2270–4. 22618382 10.1161/STROKEAHA.111.642710 PMC3404267

[CR16] Garmy-Susini B, Jin H, Zhu Y, Sung R-J, Hwang R, Varner J. Integrin α_4_β_1_-VCAM-1-mediated adhesion between endothelial and mural cells is required for blood vessel maturation. J Clin Investig. 2005;115(6):1542–51. 15902308 10.1172/JCI23445 PMC1088016

[CR17] Gosselet F, Loiola RA, Roig A, Rosell A, Culot M. Central nervous system delivery of molecules across the blood–brain barrier. Neurochem Int. 2021;144: 104952. 33400964 10.1016/j.neuint.2020.104952

[CR18] Hayakawa K, Chan SJ, Mandeville ET, Park JH, Bruzzese M, Montaner J, et al. Protective effects of endothelial progenitor cell-derived extracellular mitochondria in brain endothelium. Stem Cells. 2018;36(9):1404–10. 29781122 10.1002/stem.2856 PMC6407639

[CR19] Helms HC, Abbott NJ, Burek M, Cecchelli R, Couraud PO, Deli MA, et al. In vitro models of the blood–brain barrier: an overview of commonly used brain endothelial cell culture models and guidelines for their use. J Cereb Blood Flow Metab. 2016;36(5):862–90. 26868179 10.1177/0271678X16630991 PMC4853841

[CR20] Hossein Geranmayeh M, Farokhi-Sisakht F, Sadigh-Eteghad S, Rahbarghazi R, Mahmoudi J, Farhoudi M. Simultaneous pericytes and M2 microglia transplantation improve cognitive function in mice model of mPFC ischemia. Neuroscience. 2023;529:62–72. 37591334 10.1016/j.neuroscience.2023.08.010

[CR21] Islam MN, Das SR, Emin MT, Wei M, Sun L, Westphalen K, et al. Mitochondrial transfer from bone-marrow–derived stromal cells to pulmonary alveoli protects against acute lung injury. Nat Med. 2012;18(5):759–65. 22504485 10.1038/nm.2736 PMC3727429

[CR22] Jennings P, Koppelstaetter C, Aydin S, Abberger T, Wolf AM, Mayer G, et al. Cyclosporine A induces senescence in renal tubular epithelial cells. Am J Physiol-Renal Physiol. 2007;293(3):F831–8. 17596534 10.1152/ajprenal.00005.2007

[CR23] Kaza AK, Wamala I, Friehs I, Kuebler JD, Rathod RH, Berra I, et al. Myocardial rescue with autologous mitochondrial transplantation in a porcine model of ischemia/reperfusion. J Thorac Cardiovasc Surg. 2017;153(4):934–43. 27938904 10.1016/j.jtcvs.2016.10.077

[CR24] Khodarev NN, Roizman B, Weichselbaum RR. Molecular pathways: interferon/stat1 pathway: role in the tumor resistance to genotoxic stress and aggressive growth. Clin Cancer Res. 2012;18(11):3015–21. 22615451 10.1158/1078-0432.CCR-11-3225

[CR25] Kim I, Moon S, Hoon KS, Jin KH, Soon KY, Young KG. Vascular endothelial growth factor expression of ICAM-1, VCAM-1 and E-selectin through NFkB activation in endothelial cells. J Biol Chem. 2001;276:7614–20. 11108718 10.1074/jbc.M009705200

[CR26] Kliche S, Waltenberger J. VEGF receptor signaling and endothelial function. IUBMB Life. 2001;52(1–2):61–6. 11795595 10.1080/15216540252774784

[CR27] Kraus J, Voigt K, Schuller A, Scholz M, Kim K, Schilling M, et al. Interferon-β stabilizes barrier characteristics of the blood–brain barrier in four different species in vitro. Mult Scler J. 2008;14(6):843–52. 10.1177/1352458508088940 18505778

[CR28] Kurmann L, Okoniewski M, Dubey RK. Transcryptomic analysis of human brain-microvascular endothelial cell driven changes in-vascular pericytes. Cells. 2021;10(7):1784. 34359953 10.3390/cells10071784 PMC8304094

[CR29] Kuruganti PA, Hinojoza JR, Eaton MJ, Ehmann UK, Sobel RA. Interferon-β counteracts inflammatory mediator-induced effects on brain endothelial cell tight junction molecules—implications for multiple sclerosis. J Neuropathol Exp Neurol. 2002;61(8):710–24. 12152786 10.1093/jnen/61.8.710

[CR30] Kwon I, Kim EH, del Zoppo GJ, Heo JH. Ultrastructural and temporal changes of the microvascular basement membrane and astrocyte interface following focal cerebral ischemia. J Neurosci Res. 2009;87(3):668–76. 18831008 10.1002/jnr.21877 PMC2711693

[CR31] Lanfranconi S, Locatelli F, Corti S, Candelise L, Comi GP, Baron PL, et al. Growth factors in ischemic stroke. J Cell Mol Med. 2011;15(8):1645–87. 20015202 10.1111/j.1582-4934.2009.00987.x PMC4373358

[CR32] Lawler PR, Lawler J. Molecular basis for the regulation of angiogenesis by thrombospondin-1 and -2. Cold Spring Harb Perspect Med. 2012;2(5): a006627. 22553494 10.1101/cshperspect.a006627 PMC3331684

[CR33] Li W, Huang X, Tong H, Wang Y, Zhang T, Wang W, et al. Comparison of the regulation of β-catenin signaling by type I, type II and type III interferons in hepatocellular carcinoma cells. PLoS ONE. 2012;7(10): e47040. 23056571 10.1371/journal.pone.0047040 PMC3464253

[CR34] Lin TH, Chen Q, Howe A, Juliano RL. Cell anchorage permits efficient signal transduction between Ras and its downstream kinases. J Biol Chem. 1997;272(14):8849–52. 9082999 10.1074/jbc.272.14.8849

[CR35] Llombart V, García-Berrocoso T, Bech-Serra JJ, Simats A, Bustamante A, Giralt D, et al. Characterization of secretomes from a human blood brain barrier endothelial cells in-vitro model after ischemia by stable isotope labeling with aminoacids in cell culture (SILAC). J Proteom. 2016;133:100–12. 10.1016/j.jprot.2015.12.011 26718731

[CR36] Luo H, Gauthier M, Tan X, Landry C, Poupon JL, Dehouck M-P, et al. Sodium transporters are involved in lithium influx in brain endothelial cells. Mol Pharm. 2018;15(7):2528–38. 29874916 10.1021/acs.molpharmaceut.8b00018

[CR37] Ma Y, Zechariah A, Qu Y, Hermann DM. Effects of vascular endothelial growth factor in ischemic stroke. J Neurosci Res. 2012;90(10):1873–82. 22714747 10.1002/jnr.23088

[CR38] Ma F, Martinez-San SP, Barcelo V, Morancho A, Gabriel-Salazar M, Giralt D, et al. Matrix metalloproteinase-13 participates in neuroprotection and neurorepair after cerebral ischemia in mice. Neurobiol Dis. 2016;91:236–46. 27001146 10.1016/j.nbd.2016.03.016

[CR39] Maddahi A, Edvinsson L. Cerebral ischemia induces microvascular pro-inflammatory cytokine expression via the MEK/ERK pathway. J Neuroinflamm. 2010;7(1):1–13. 10.1186/1742-2094-7-14 PMC2837637 20187933

[CR40] Maki T, Morancho A, Martinez-San SP, Hayakawa K, Takase H, Liang AC, et al. Endothelial progenitor cell secretome and oligovascular repair in a mouse model of prolonged cerebral hypoperfusion. Stroke. 2018;49(4):1003–10. 29511131 10.1161/STROKEAHA.117.019346 PMC5871569

[CR41] Marjolein Heymans RF, Dehouck L, Francisco D, Sano Y, Shimizu F, Kanda T, Bruggmann R, Engelhardt B, Winter P, Gosselet F, Culot M. Contribution of brain pericytes in blood–brain barrier formation and maintenance: a transcriptomic study of cocultured human endothelial cells derived from hematopoietic stem cells. Fluids Barriers CNS. 2020;17(1):48. 32723387 10.1186/s12987-020-00208-1 PMC7385894

[CR42] Melander E, Eriksson C, Wellens S, Hosseini K, Fredriksson R, Gosselet F, et al. Differential blood–brain barrier transport and cell uptake of cyclic peptides in vivo and in vitro. Pharmaceutics. 2023;15(5):1507. 37242750 10.3390/pharmaceutics15051507 PMC10222203

[CR43] Montesano R, Orci L, Vassalli P. In vitro rapid organization of endothelial cells into capillary-like networks is promoted by collagen matrices. J Cell Biol. 1983;97(5 Pt 1):1648–52. 6630296 10.1083/jcb.97.5.1648 PMC2112683

[CR44] Morancho A, Hernandez-Guillamon M, Boada C, Barcelo V, Giralt D, Ortega L, et al. Cerebral ischaemia and matrix metalloproteinase-9 modulate the angiogenic function of early and late outgrowth endothelial progenitor cells. J Cell Mol Med. 2013;17(12):1543–53. 23945132 10.1111/jcmm.12116 PMC3914647

[CR45] Morancho A, Ma F, Barcelo V, Giralt D, Montaner J, Rosell A. Impaired vascular remodeling after endothelial progenitor cell transplantation in MMP9-deficient mice suffering cortical cerebral ischemia. J Cereb Blood Flow Metab. 2015;35(10):1547–51. 26219597 10.1038/jcbfm.2015.180 PMC4640313

[CR46] Narasimhan P, Liu J, Song YS, Massengale JL, Chan PH. VEGF stimulates the ERK 1/2 signaling pathway and apoptosis in cerebral endothelial cells after ischemic conditions. Stroke. 2009;40(4):1467–73. 19228841 10.1161/STROKEAHA.108.534644 PMC2663599

[CR47] Nistor-Cseppentö DC, Jurcău MC, Jurcău A, Andronie-Cioară FL, Marcu F. Stem cell- and cell-based therapies for ischemic stroke. Bioengineering. 2022;9(11):717. 36421118 10.3390/bioengineering9110717 PMC9687728

[CR48] Pedroso DC, Tellechea A, Moura L, Fidalgo-Carvalho I, Duarte J, Carvalho E, et al. Improved survival, vascular differentiation and wound healing potential of stem cells co-cultured with endothelial cells. PLoS ONE. 2011;6(1): e16114. 21283630 10.1371/journal.pone.0016114 PMC3026015

[CR49] Prabhakaran S, Ruff I, Bernstein RA. Acute stroke intervention: a systematic review. JAMA. 2015;313(14):1451–62. 25871671 10.1001/jama.2015.3058

[CR50] Rendon-Mitchell B, Ochani M, Li J, Han J, Wang H, Yang H, et al. IFN-gamma induces high mobility group box 1 protein release partly through a TNF-dependent mechanism. J Immunol. 2003;170(7):3890–7. 12646658 10.4049/jimmunol.170.7.3890

[CR51] Rosell A, Morancho A, Navarro-Sobrino M, Martínez-Saez E, Hernández-Guillamon M, Lope-Piedrafita S, et al. Factors secreted by endothelial progenitor cells enhance neurorepair responses after cerebral ischemia in mice. PLoS ONE. 2013;8(9): e73244. 24023842 10.1371/journal.pone.0073244 PMC3762828

[CR52] Saint-Pol J, Gosselet F. Oxysterols and the neurovascular unit (NVU): a far true love with bright and dark sides. J Steroid Biochem Mol Biol. 2019;191: 105368. 31026511 10.1016/j.jsbmb.2019.04.017

[CR53] Saint-Pol J, Gosselet F, Duban-Deweer S, Pottiez G, Karamanos Y. Targeting and crossing the blood–brain barrier with extracellular vesicles. Cells. 2020;9(4):851. 32244730 10.3390/cells9040851 PMC7226770

[CR54] Shawahna R, Ganeshamoorthy K, Huilong L, Scherrmann JM, Couraud PO, Declèves X. Effect of long-term in vitro lithium exposure on mRNA levels of claudin-3, CYP1A1, ABCG2 and GSTM3 Genes in the hCMEC/D3 human brain endothelial cell line. Eur J Drug Metab Pharmacokinet. 2017;42(6):1013–7. 28367588 10.1007/s13318-017-0412-3

[CR55] Shimizu F, Sano Y, Abe MA, Maeda T, Ohtsuki S, Terasaki T, et al. Peripheral nerve pericytes modify the blood–nerve barrier function and tight junctional molecules through the secretion of various soluble factors. J Cell Physiol. 2011;226(1):255–66. 20665675 10.1002/jcp.22337

[CR56] Smith JL, Jeng S, McWeeney SK, Hirsch AJ. A microRNA screen identifies the Wnt signaling pathway as a regulator of the interferon response during flavivirus infection. J Virol. 2017;91(8):e02388-e12316. 28148804 10.1128/JVI.02388-16 PMC5375670

[CR57] Soldi R, Mitola S, Strasly M, Defilippi P, Tarone G, Bussolino F. Role of alphavbeta3 integrin in the activation of vascular endothelial growth factor receptor-2. EMBO J. 1999;18(4):882–92. 10022831 10.1093/emboj/18.4.882 PMC1171181

[CR58] Taguchi A, Soma T, Tanaka H, Kanda T, Nishimura H, Yoshikawa H, et al. Administration of CD34+ cells after stroke enhances neurogenesis via angiogenesisin a mouse model. J Clin Investig. 2004;114(3):330–8. 15286799 10.1172/JCI200420622 PMC484977

[CR59] Thompson MD, Dar MJ, Monga SP. Pegylated interferon alpha targets Wnt signaling by inducing nuclear export of β-catenin. J Hepatol. 2011;54(3):506–12. 21093092 10.1016/j.jhep.2010.07.020 PMC3052972

[CR60] Uchida Y, Ohtsuki S, Katsukura Y, Ikeda C, Suzuki T, Kamiie J, et al. Quantitative targeted absolute proteomics of human blood–brain barrier transporters and receptors. J Neurochem. 2011;117(2):333–45. 21291474 10.1111/j.1471-4159.2011.07208.x

[CR61] Veldhuis WB, Derksen JW, Floris S, Van Der Meide PH, De Vries HE, Schepers J, et al. Interferon-beta blocks infiltration of inflammatory cells and reduces infarct volume after ischemic stroke in the rat. J Cereb Blood Flow Metab. 2003;23(9):1029–39. 12973019 10.1097/01.WCB.0000080703.47016.B6

[CR62] Wechsler LR, Bates D, Stroemer P, Andrews-Zwilling YS, Aizman I. Cell therapy for chronic stroke. Stroke. 2018;49(5):1066–74. 29669865 10.1161/STROKEAHA.117.018290

[CR63] Weksler BB, Subileau EA, Perriere N, Charneau P, Holloway K, Leveque M, et al. Blood–brain barrier-specific properties of a human adult brain endothelial cell line. FASEB J. 2005;19(13):1872–4. 16141364 10.1096/fj.04-3458fje

[CR64] Wellens S, Dehouck L, Chandrasekaran V, Singh P, Loiola RA, Sevin E, et al. Evaluation of a human iPSC-derived BBB model for repeated dose toxicity testing with cyclosporine A as model compound. Toxicol in Vitro. 2021;73: 105112. 33631201 10.1016/j.tiv.2021.105112

[CR65] West XZ, Meller N, Malinin NL, Deshmukh L, Meller J, Mahabeleshwar GH, et al. Integrin β 3 crosstalk with VEGFR accommodating tyrosine phosphorylation as a regulatory switch. PLoS ONE. 2012;7(2): e31071. 22363548 10.1371/journal.pone.0031071 PMC3281915

[CR66] Wu G, Luo J, Rana JS, Laham R, Sellke FW, Li J. Involvement of COX-2 in VEGF-induced angiogenesis via P38 and JNK pathways in vascular endothelial cells. Cardiovasc Res. 2006;69(2):512–9. 16336951 10.1016/j.cardiores.2005.09.019

[CR67] Xu X, Chai K, Chen Y, Lin Y, Zhang S, Li X, et al. Interferon activates promoter of Nmi gene via interferon regulator factor-1. Mol Cell Biochem. 2018;441(1–2):165–71. 28913576 10.1007/s11010-017-3182-y

[CR68] Zhao C, Collins MN, Hsiang TY, Krug RM. Interferon-induced ISG15 pathway: an ongoing virus-host battle. Trends Microbiol. 2013;21(4):181–6. 23414970 10.1016/j.tim.2013.01.005 PMC3622817

