# Supplementary material for: Secretome of brain microvascular endothelial cells promotes endothelial barrier tightness and protects against hypoxia-induced vascular leakage
Source: Mol Med. 2024 Aug 26;30:132. doi: 10.1186/s10020-024-00897-6 (PMC11348522; doi:10.1186/s10020-024-00897-6)
Supplement: Supplementary file 1 — Supplementary Figure 1. Representative images of pilot experiments performed to test the antibodies used in the study. Black arrows show the corresponding bands for every antibody according to the molecular weight predicted by the manufacturer. (a) phospho-AKT and pan-AKT; (b) phospho-VEGFR2 and pan-VEGFR2; (c) phospho-ERK1/2 and pan-ERK 1/2; (d) non-phospho-β-catenin and pan-β-catenin; (e) ABCA1; (f) BCRP; (g) P-gp; (h) COX-2; (i) VCAM-1; (j) ICAM-1; (k) claudin 5; (l) VE-cadherin; (m) occludin; (n) ZO-1; (o) tricellulin; (p) claudin 3. For images A–D, membranes were firstly probed with phospho-antibodies and then reprobed with pan-antibodies. [file 10020_2024_897_MOESM1_ESM.pptx]

## Slide 1
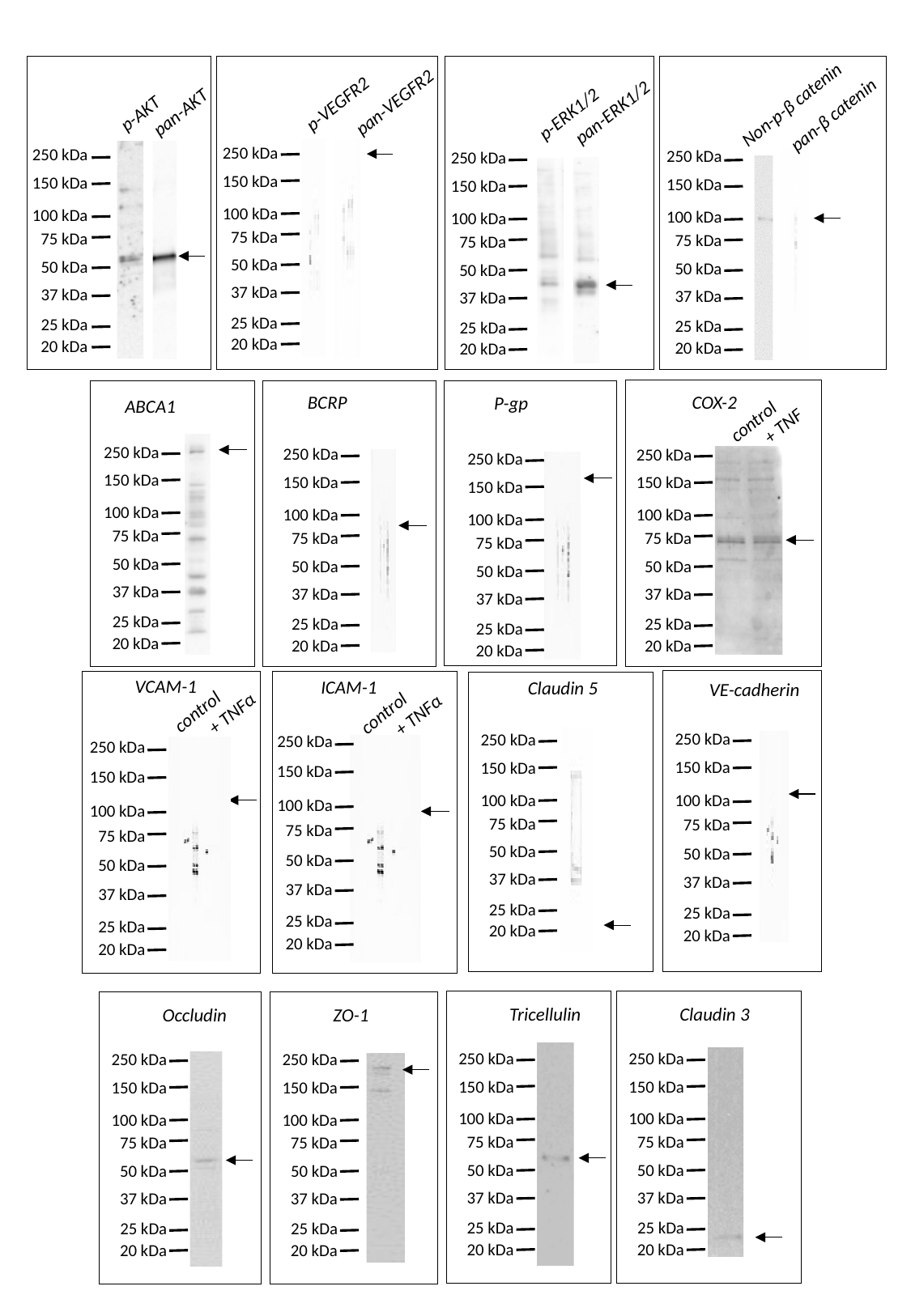

a
b
c
pan-VEGFR2
p-VEGFR2
250 kDa
150 kDa
100 kDa
75 kDa
50 kDa
37 kDa
25 kDa
20 kDa
d
Non-p-β catenin
pan-AKT
pan-ERK1/2
p-AKT
p-ERK1/2
pan-β catenin
250 kDa
250 kDa
150 kDa
100 kDa
75 kDa
50 kDa
37 kDa
25 kDa
20 kDa
250 kDa
150 kDa
100 kDa
75 kDa
50 kDa
37 kDa
25 kDa
20 kDa
150 kDa
100 kDa
75 kDa
50 kDa
37 kDa
25 kDa
20 kDa
e
f
g
h
COX-2
BCRP
P-gp
ABCA1
control
+ TNF
250 kDa
150 kDa
100 kDa
75 kDa
50 kDa
37 kDa
25 kDa
20 kDa
250 kDa
150 kDa
100 kDa
75 kDa
50 kDa
37 kDa
25 kDa
20 kDa
250 kDa
150 kDa
100 kDa
75 kDa
50 kDa
37 kDa
25 kDa
20 kDa
250 kDa
150 kDa
100 kDa
75 kDa
50 kDa
37 kDa
25 kDa
20 kDa
VCAM-1
control
+ TNFα
250 kDa
150 kDa
100 kDa
75 kDa
50 kDa
37 kDa
25 kDa
20 kDa
k
ICAM-1
control
+ TNFα
250 kDa
150 kDa
100 kDa
75 kDa
50 kDa
37 kDa
25 kDa
20 kDa
j
Claudin 5
i
l
VE-cadherin
250 kDa
150 kDa
100 kDa
75 kDa
50 kDa
37 kDa
25 kDa
20 kDa
250 kDa
150 kDa
100 kDa
75 kDa
50 kDa
37 kDa
25 kDa
20 kDa
n
o
p
m
Tricellulin
Claudin 3
Occludin
ZO-1
250 kDa
150 kDa
100 kDa
75 kDa
50 kDa
37 kDa
25 kDa
20 kDa
250 kDa
150 kDa
100 kDa
75 kDa
50 kDa
37 kDa
25 kDa
20 kDa
250 kDa
150 kDa
100 kDa
75 kDa
50 kDa
37 kDa
25 kDa
20 kDa
250 kDa
150 kDa
100 kDa
75 kDa
50 kDa
37 kDa
25 kDa
20 kDa
