# Supplementary material for: Secretome of brain microvascular endothelial cells promotes endothelial barrier tightness and protects against hypoxia-induced vascular leakage
Source: Mol Med. 2024 Aug 26;30:132. doi: 10.1186/s10020-024-00897-6 (PMC11348522; doi:10.1186/s10020-024-00897-6)
Supplement: Supplementary file 2 — Supplementary Figure 2. Images used for western blotting analysis of ERK1/2, AKT, and VEGFR2 phosphorylation in proliferative CD34+-EC in response to acute administration (0–60 min) of scHSP (5 μg/mL) (Fig. 3a). bFGF (basic FGF) represented in (a) was not considered for analysis. [file 10020_2024_897_MOESM2_ESM.pptx]

## Slide 1
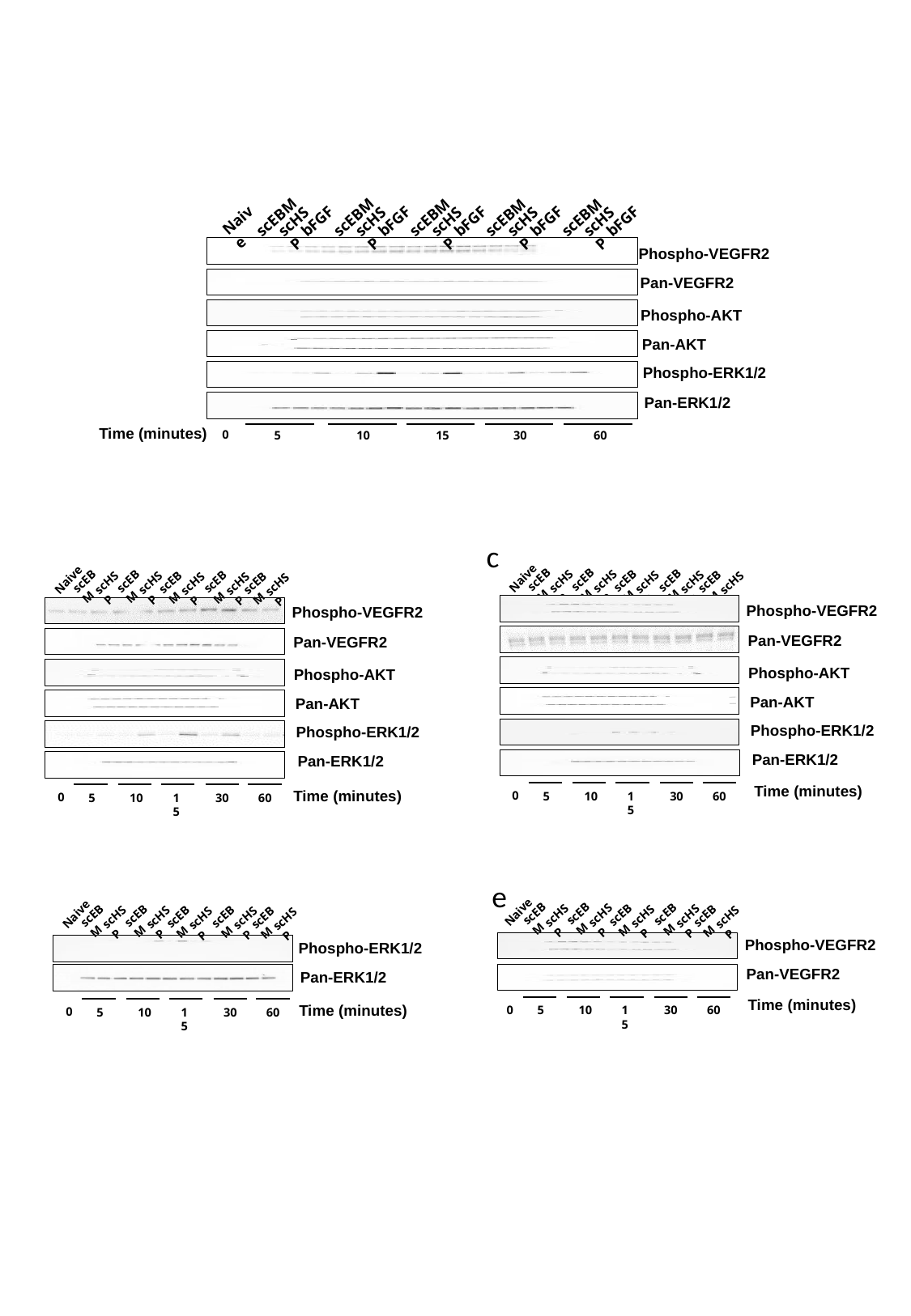

a
scEBM
scHSP
bFGF
scEBM
scHSP
bFGF
scEBM
scHSP
bFGF
scEBM
scHSP
bFGF
scEBM
scHSP
bFGF
Naive
Phospho-VEGFR2
Pan-VEGFR2
Phospho-AKT
Pan-AKT
Phospho-ERK1/2
Pan-ERK1/2
Time (minutes)
0
5
10
15
30
60
b
c
Naive
scEBM
scHSP
scEBM
scHSP
scEBM
scHSP
scEBM
scHSP
scEBM
scHSP
Phospho-VEGFR2
Pan-VEGFR2
Phospho-AKT
Pan-AKT
Phospho-ERK1/2
Pan-ERK1/2
0
5
10
15
30
60
Time (minutes)
Naive
scEBM
scHSP
scEBM
scHSP
scEBM
scHSP
scEBM
scHSP
scEBM
scHSP
Phospho-VEGFR2
Pan-VEGFR2
Phospho-AKT
Pan-AKT
Phospho-ERK1/2
Pan-ERK1/2
0
5
10
15
30
60
Time (minutes)
d
e
Naive
scEBM
scHSP
scEBM
scHSP
scEBM
scHSP
scEBM
scHSP
scEBM
scHSP
Phospho-VEGFR2
Pan-VEGFR2
0
5
10
15
30
60
Time (minutes)
Naive
scEBM
scHSP
scEBM
scHSP
scEBM
scHSP
scEBM
scHSP
scEBM
scHSP
Phospho-ERK1/2
Pan-ERK1/2
0
5
10
15
30
60
Time (minutes)
