# Supplementary material for: Secretome of brain microvascular endothelial cells promotes endothelial barrier tightness and protects against hypoxia-induced vascular leakage
Source: Mol Med. 2024 Aug 26;30:132. doi: 10.1186/s10020-024-00897-6 (PMC11348522; doi:10.1186/s10020-024-00897-6)
Supplement: Supplementary file 3 — Supplementary Figure 3. Images used for western blotting analysis of ERK1/2, AKT, and VEGFR2 phosphorylation in proliferative CD34+-EC in response to administration (4 or 24 h) of scHSP (5 μg/mL) (Fig. 3b). [file 10020_2024_897_MOESM3_ESM.pptx]

## Slide 1
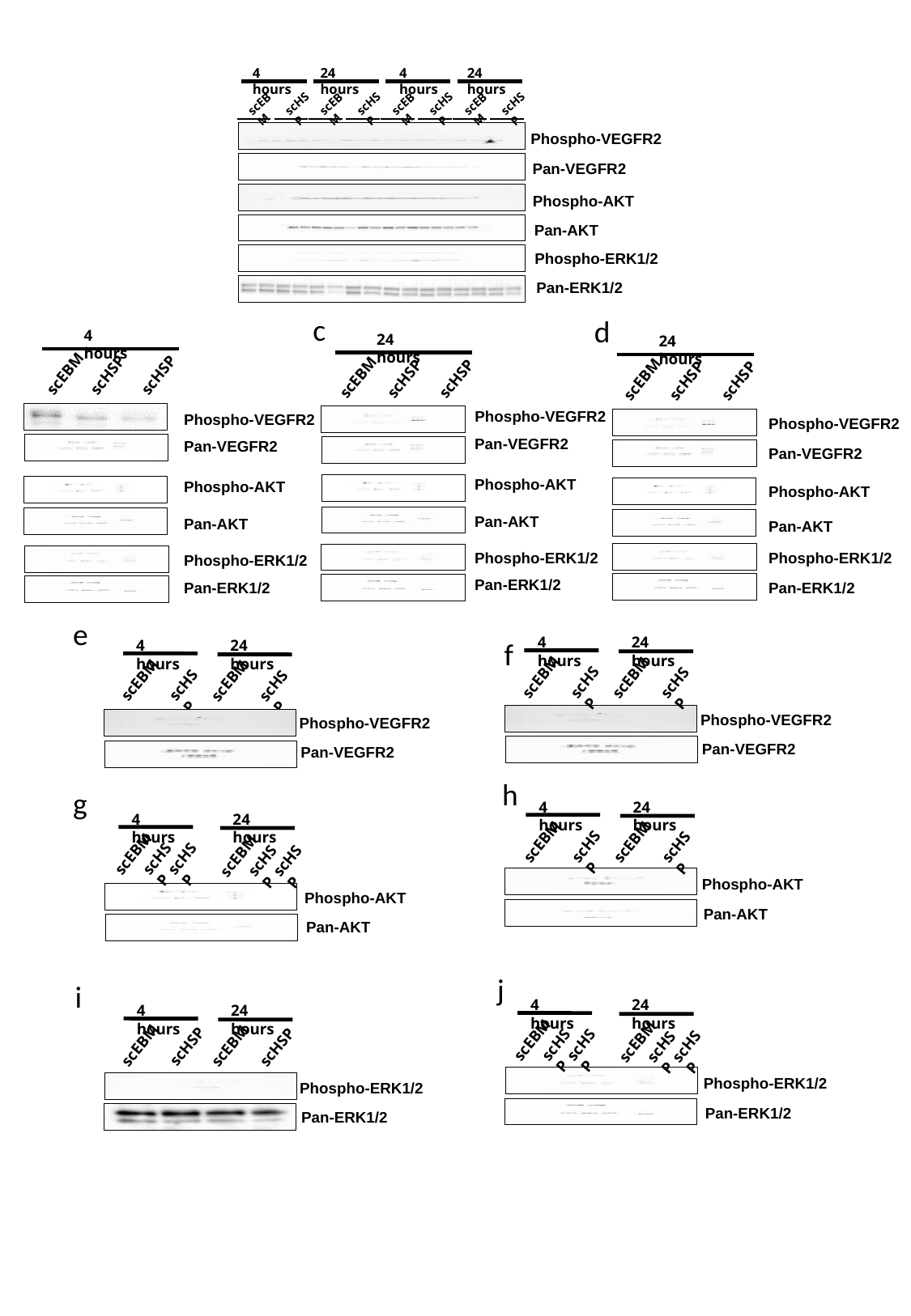

a
4 hours
24 hours
4 hours
24 hours
scEBM
scHSP
scEBM
scHSP
scEBM
scHSP
scEBM
scHSP
Phospho-VEGFR2
Pan-VEGFR2
Phospho-AKT
Pan-AKT
Phospho-ERK1/2
Pan-ERK1/2
scEBM
scHSP
scHSP
4 hours
Phospho-VEGFR2
Pan-VEGFR2
Phospho-AKT
Pan-AKT
Phospho-ERK1/2
Pan-ERK1/2
scEBM
scHSP
scHSP
24 hours
Phospho-VEGFR2
Pan-VEGFR2
Phospho-AKT
Pan-AKT
Phospho-ERK1/2
Pan-ERK1/2
scEBM
scHSP
scHSP
24 hours
Phospho-VEGFR2
Pan-VEGFR2
Phospho-AKT
Pan-AKT
Phospho-ERK1/2
Pan-ERK1/2
b
c
d
e
4 hours
24 hours
scEBM
scHSP
scEBM
scHSP
Phospho-VEGFR2
Pan-VEGFR2
f
4 hours
24 hours
scEBM
scHSP
scEBM
scHSP
Phospho-VEGFR2
Pan-VEGFR2
h
g
4 hours
24 hours
scEBM
scHSP
scEBM
scHSP
Phospho-AKT
Pan-AKT
4 hours
24 hours
scEBM
scHSP
scHSP
scEBM
scHSP
scHSP
Phospho-AKT
Pan-AKT
j
i
4 hours
24 hours
scEBM
scHSP
scHSP
scEBM
scHSP
scHSP
Phospho-ERK1/2
Pan-ERK1/2
4 hours
24 hours
scHSP
scEBM
scHSP
scEBM
Phospho-ERK1/2
Pan-ERK1/2
