# Supplementary material for: Secretome of brain microvascular endothelial cells promotes endothelial barrier tightness and protects against hypoxia-induced vascular leakage
Source: Mol Med. 2024 Aug 26;30:132. doi: 10.1186/s10020-024-00897-6 (PMC11348522; doi:10.1186/s10020-024-00897-6)
Supplement: Supplementary file 4 — Supplementary Figure 4. Images used for western blotting analysis of ZO-1, VE-cadherin, occludin, claudin 5, claudin 3, non-phospho-catenin, and pan-catenin in CMECs in response to administration (48 h) of scHSP (5 μg/mL) (Fig. 4b, e). scHSP-1: batch 1; scHSP-2: batch 2. Analysis of scHSP-2 were not presented in the manuscript. [file 10020_2024_897_MOESM4_ESM.pptx]

## Slide 1
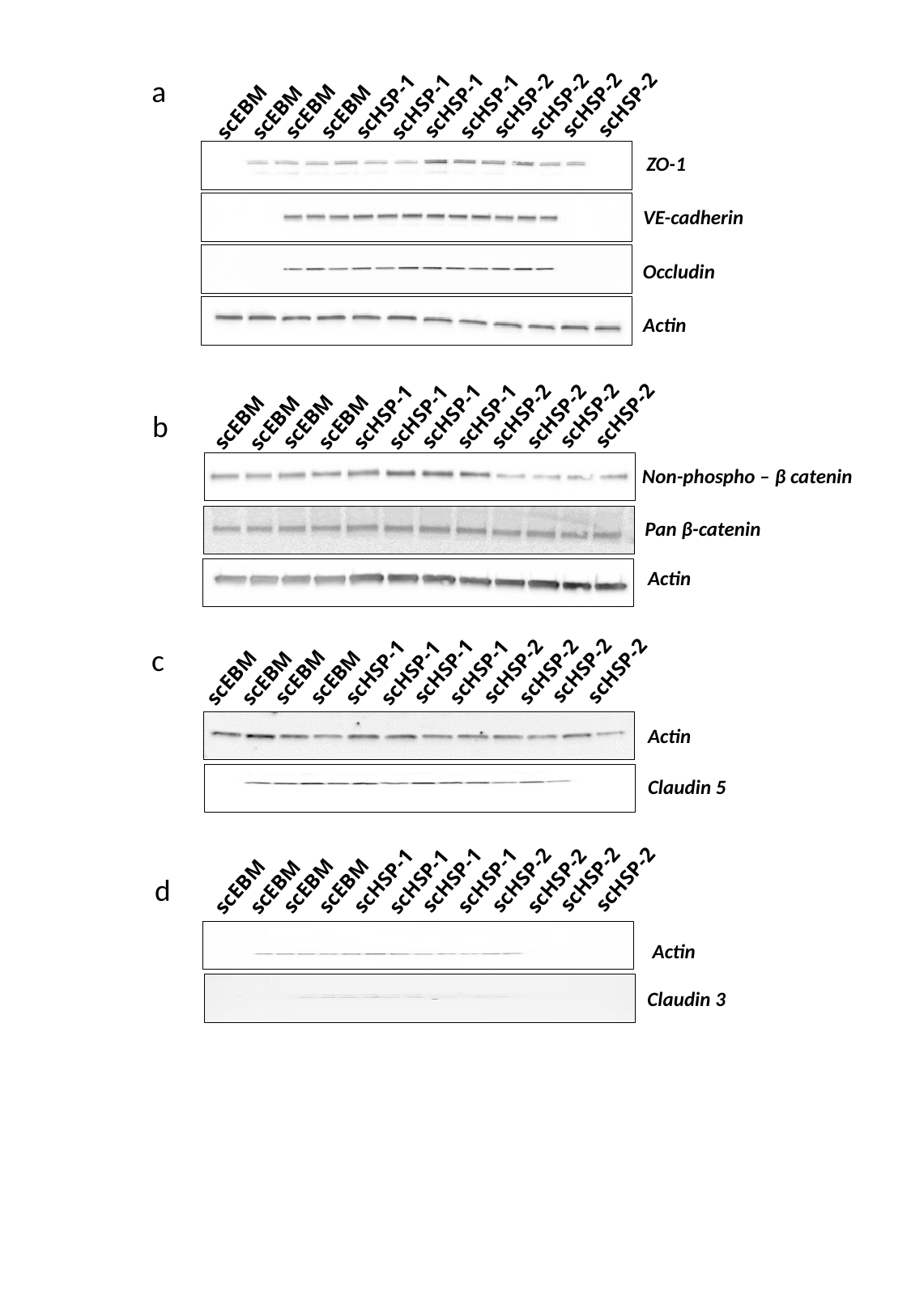

scHSP-2
scHSP-2
scHSP-2
scHSP-2
scHSP-1
scHSP-1
scHSP-1
scHSP-1
scEBM
scEBM
scEBM
scEBM
a
ZO-1
VE-cadherin
Occludin
Actin
scHSP-2
scHSP-2
scHSP-2
scHSP-2
scHSP-1
scHSP-1
scHSP-1
scHSP-1
scEBM
scEBM
scEBM
scEBM
b
Non-phospho – β catenin
Pan β-catenin
Actin
scHSP-2
scHSP-2
scHSP-2
scHSP-2
scHSP-1
scHSP-1
scHSP-1
scHSP-1
scEBM
scEBM
scEBM
scEBM
c
Actin
Claudin 5
scHSP-2
scHSP-2
scHSP-2
scHSP-2
scHSP-1
scHSP-1
scHSP-1
scHSP-1
scEBM
scEBM
scEBM
scEBM
d
Actin
Claudin 3
