# Supplementary material for: Secretome of brain microvascular endothelial cells promotes endothelial barrier tightness and protects against hypoxia-induced vascular leakage
Source: Mol Med. 2024 Aug 26;30:132. doi: 10.1186/s10020-024-00897-6 (PMC11348522; doi:10.1186/s10020-024-00897-6)
Supplement: Supplementary file 6 — Supplementary Figure 6. Images used for western blotting analysis claudin 5, tricellulin, VEGFR2, VCAM-1, COX-2, occludin, VE-Cadherin, ZO-1, and ERK1/2 in CMECs exposed to normoxic (N) or OGD conditions and reoxygenation (R-N-scEBM; R-N-scHSP; R-OGD-scEBM; R-OGD-scHSP) (Figs. 5 and supplementary Fig. 15). [file 10020_2024_897_MOESM6_ESM.pptx]

## Slide 1
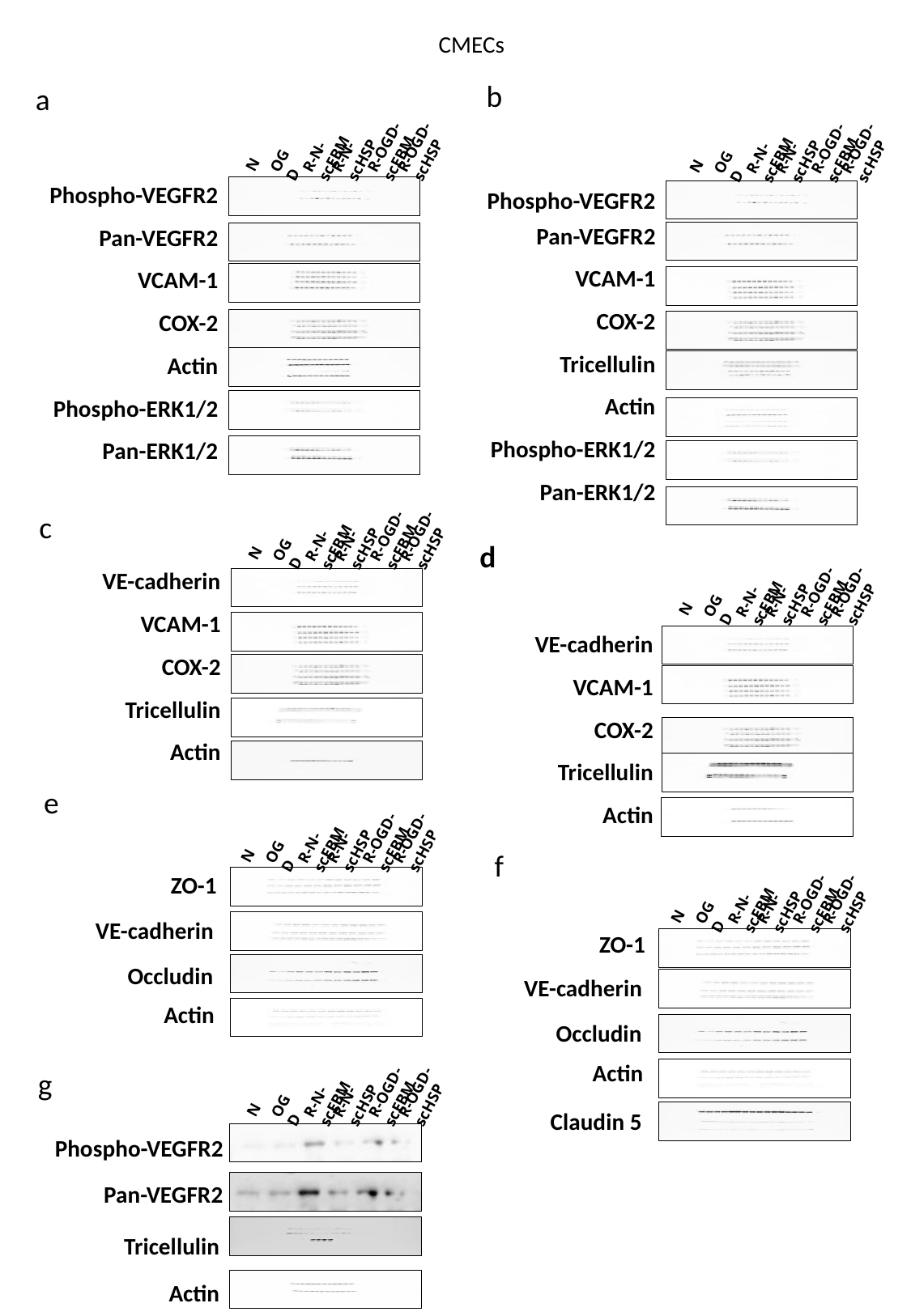

CMECs
R-OGD-scEBM
R-OGD-scHSP
R-N-scEBM
R-N-scHSP
OGD
N
Phospho-VEGFR2
Pan-VEGFR2
VCAM-1
COX-2
Actin
Phospho-ERK1/2
Pan-ERK1/2
b
R-OGD-scEBM
R-OGD-scHSP
R-N-scEBM
R-N-scHSP
OGD
N
Phospho-VEGFR2
Pan-VEGFR2
VCAM-1
COX-2
Tricellulin
Actin
Phospho-ERK1/2
Pan-ERK1/2
a
R-OGD-scEBM
R-OGD-scHSP
R-N-scEBM
R-N-scHSP
OGD
N
VE-cadherin
VCAM-1
COX-2
Tricellulin
Actin
c
R-OGD-scEBM
R-OGD-scHSP
R-N-scEBM
R-N-scHSP
OGD
N
VCAM-1
Tricellulin
d
VE-cadherin
COX-2
Actin
R-OGD-scEBM
R-OGD-scHSP
R-N-scEBM
R-N-scHSP
OGD
N
ZO-1
VE-cadherin
Occludin
Actin
e
R-OGD-scEBM
R-OGD-scHSP
R-N-scEBM
R-N-scHSP
OGD
N
ZO-1
VE-cadherin
Occludin
Actin
Claudin 5
f
R-OGD-scEBM
R-OGD-scHSP
R-N-scEBM
R-N-scHSP
OGD
N
Phospho-VEGFR2
Pan-VEGFR2
Tricellulin
Actin
g
