# Supplementary material for: Secretome of brain microvascular endothelial cells promotes endothelial barrier tightness and protects against hypoxia-induced vascular leakage
Source: Mol Med. 2024 Aug 26;30:132. doi: 10.1186/s10020-024-00897-6 (PMC11348522; doi:10.1186/s10020-024-00897-6)
Supplement: Supplementary file 7 — Supplementary Figure 7. Images used for western blotting analysis of claudin 5, ZO-1, VE-cadherin, VEGFR2, VCAM, AKT, ERK1/2, and occludin in CMECs exposed to normoxic (N) or OGD conditions and reoxygenation (R-N-scEBM; R-N-scHSP; R-OGD-scEBM; R-OGD-scHSP) (Fig. 5 and supplementary Fig. 15). [file 10020_2024_897_MOESM7_ESM.pptx]

## Slide 1
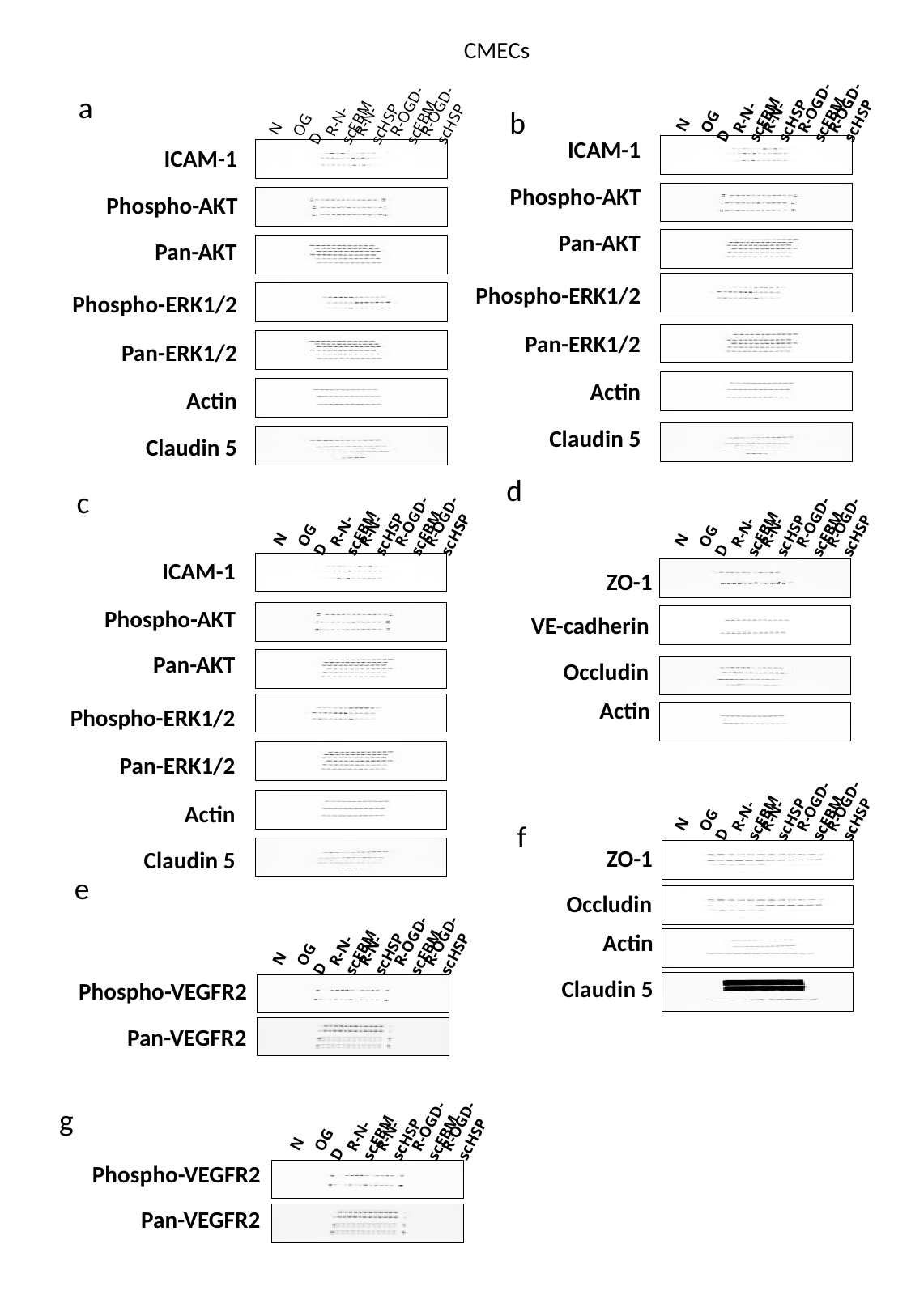

CMECs
R-OGD-scEBM
R-OGD-scHSP
R-N-scEBM
R-N-scHSP
OGD
N
ICAM-1
Phospho-AKT
Pan-AKT
Phospho-ERK1/2
Pan-ERK1/2
Actin
Claudin 5
R-OGD-scEBM
R-OGD-scHSP
R-N-scEBM
R-N-scHSP
OGD
N
a
b
ICAM-1
Phospho-AKT
Pan-AKT
Phospho-ERK1/2
Pan-ERK1/2
Actin
Claudin 5
R-OGD-scEBM
R-OGD-scHSP
R-N-scEBM
R-N-scHSP
OGD
N
ICAM-1
Phospho-AKT
Pan-AKT
Phospho-ERK1/2
Pan-ERK1/2
Actin
Claudin 5
R-OGD-scEBM
R-OGD-scHSP
R-N-scEBM
R-N-scHSP
OGD
N
ZO-1
VE-cadherin
Occludin
Actin
d
c
R-OGD-scEBM
R-OGD-scHSP
R-N-scEBM
R-N-scHSP
OGD
N
ZO-1
Occludin
Actin
Claudin 5
f
e
R-OGD-scEBM
R-OGD-scHSP
R-N-scEBM
R-N-scHSP
OGD
N
Phospho-VEGFR2
Pan-VEGFR2
R-OGD-scEBM
R-OGD-scHSP
R-N-scEBM
R-N-scHSP
OGD
N
Phospho-VEGFR2
Pan-VEGFR2
g
