# Supplementary material for: Secretome of brain microvascular endothelial cells promotes endothelial barrier tightness and protects against hypoxia-induced vascular leakage
Source: Mol Med. 2024 Aug 26;30:132. doi: 10.1186/s10020-024-00897-6 (PMC11348522; doi:10.1186/s10020-024-00897-6)
Supplement: Supplementary file 10 — Supplementary Figure 10. Images used for western blotting analysis of ABCA1, BCRP, and P-gp in CMECs (a–c) and BLECs (d, e) in response to administration (48 h) of scHSP (5 μg/mL) (Supplementary Fig. 14c). scHSP-1: batch 1; scHSP-2: batch 2. Analysis of scHSP-2 were not presented in the manuscript. [file 10020_2024_897_MOESM10_ESM.pptx]

## Slide 1
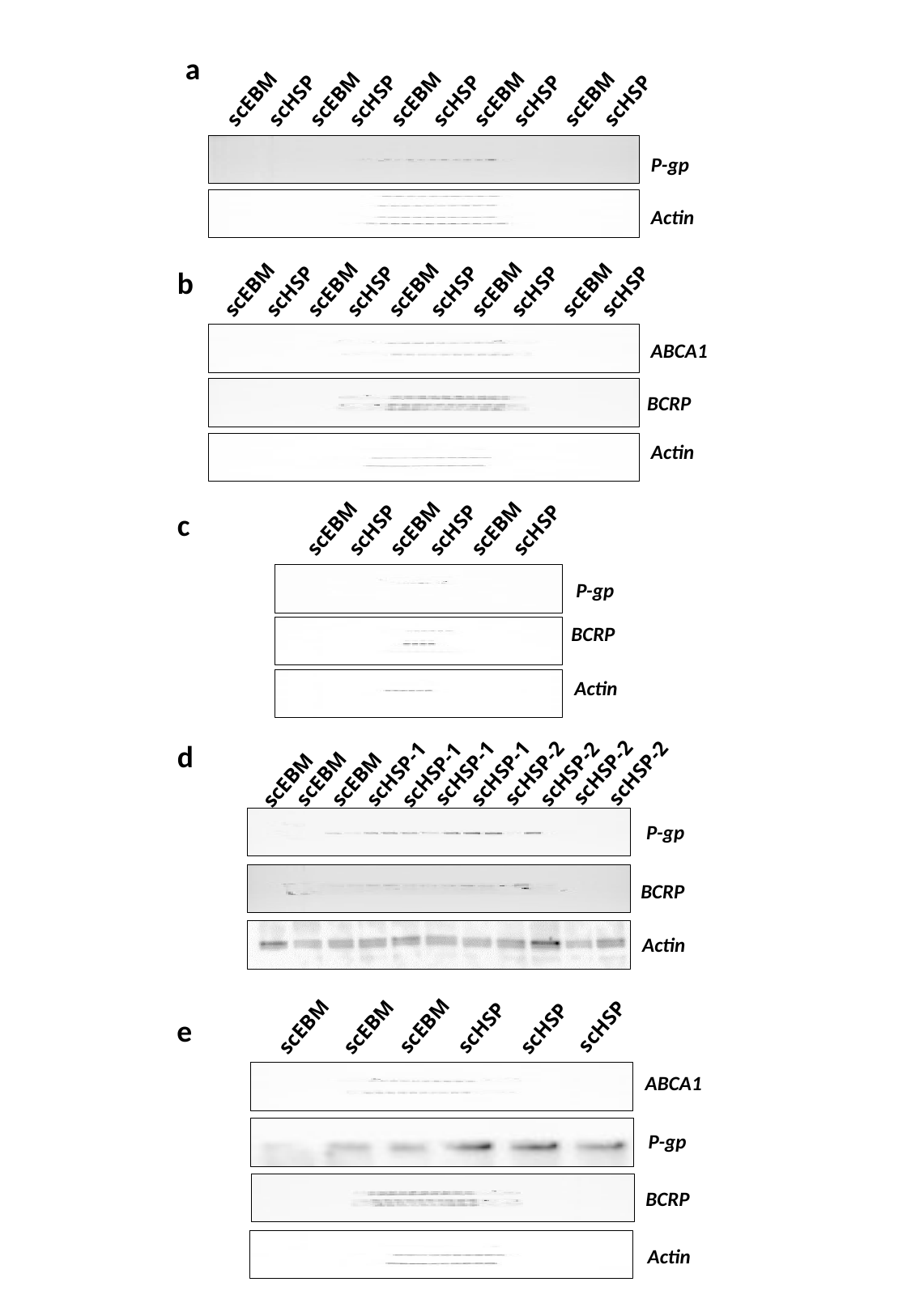

a
scEBM
scHSP
scEBM
scHSP
scEBM
scHSP
scEBM
scHSP
scEBM
scHSP
P-gp
Actin
b
scEBM
scHSP
scEBM
scHSP
scEBM
scHSP
scEBM
scHSP
scEBM
scHSP
ABCA1
BCRP
Actin
scEBM
scHSP
scEBM
scHSP
scEBM
scHSP
P-gp
BCRP
Actin
c
scHSP-2
scHSP-2
scHSP-2
scHSP-2
scHSP-1
scHSP-1
scHSP-1
scHSP-1
scEBM
scEBM
scEBM
d
P-gp
BCRP
Actin
scHSP
scHSP
scHSP
scEBM
scEBM
scEBM
e
ABCA1
P-gp
BCRP
Actin
