# Supplementary material for: Secretome of brain microvascular endothelial cells promotes endothelial barrier tightness and protects against hypoxia-induced vascular leakage
Source: Mol Med. 2024 Aug 26;30:132. doi: 10.1186/s10020-024-00897-6 (PMC11348522; doi:10.1186/s10020-024-00897-6)
Supplement: Supplementary file 11 — Supplementary Figure 11. Images used for western blotting analysis of claudin 5, ZO-1, occludin, and VE-cadherin in BLECs treated with vehicle or TNFα (10 ng/mL) (Supplementary Fig. 16c). [file 10020_2024_897_MOESM11_ESM.pptx]

## Slide 1
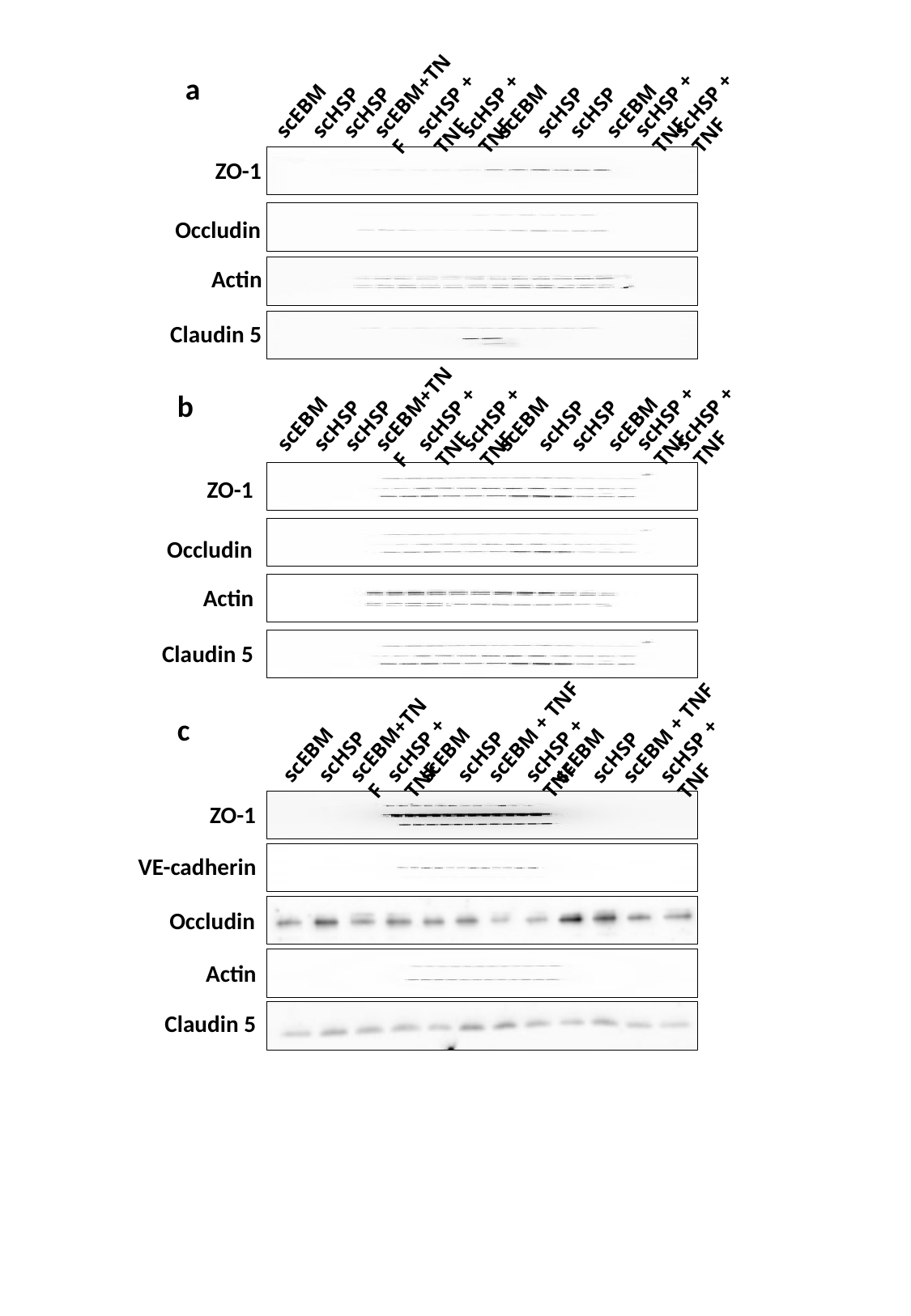

a
scHSP + TNF
scHSP + TNF
scEBM+TNF
scHSP + TNF
scHSP + TNF
scEBM
scHSP
scHSP
scEBM
scHSP
scHSP
scEBM
ZO-1
Occludin
Actin
Claudin 5
b
scHSP + TNF
scHSP + TNF
scEBM+TNF
scHSP + TNF
scHSP + TNF
scEBM
scHSP
scHSP
scEBM
scHSP
scHSP
scEBM
ZO-1
Occludin
Actin
Claudin 5
c
scEBM + TNF
scEBM + TNF
scHSP + TNF
scEBM+TNF
scHSP + TNF
scHSP + TNF
scEBM
scHSP
scEBM
scHSP
scEBM
scHSP
ZO-1
VE-cadherin
Occludin
Actin
Claudin 5
