# Supplementary figures and images for: Secretome of brain microvascular endothelial cells promotes endothelial barrier tightness and protects against hypoxia-induced vascular leakage
Source: Mol Med. 2024 Aug 26;30:132. doi: 10.1186/s10020-024-00897-6 (PMC11348522; doi:10.1186/s10020-024-00897-6)

## Slide 1
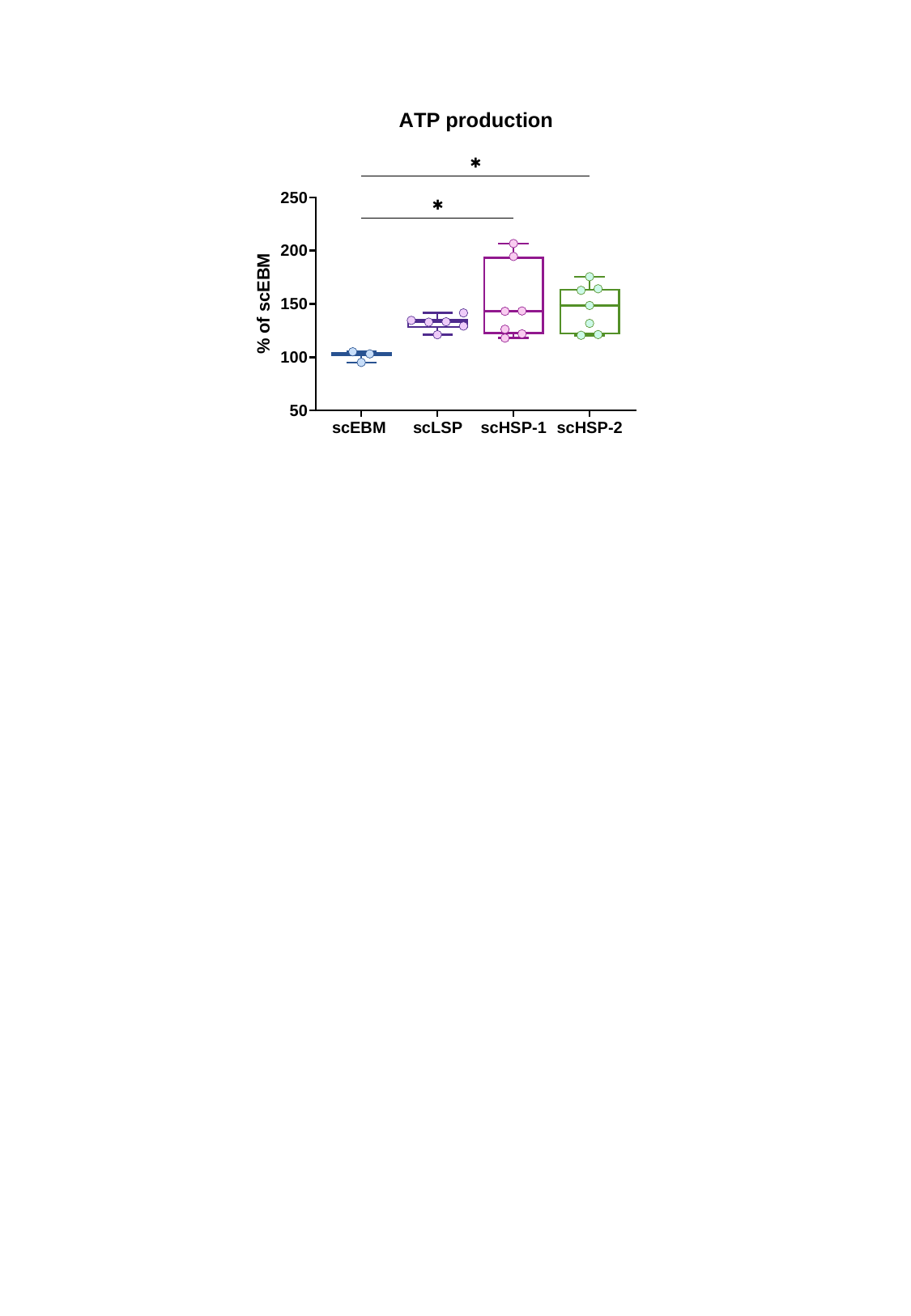

Supplement: Supplementary file 12 — Supplementary Figure 12. scHSP increases CD34+-ECs proliferation. scLSP (batches 1 to 6), and scHSP (batches 1 and 2) increases ATP production by CD34+-ECs. Data represents median (interquartile range), Kruskal–Wallis test, versus scEBM. [file 10020_2024_897_MOESM12_ESM.pptx]

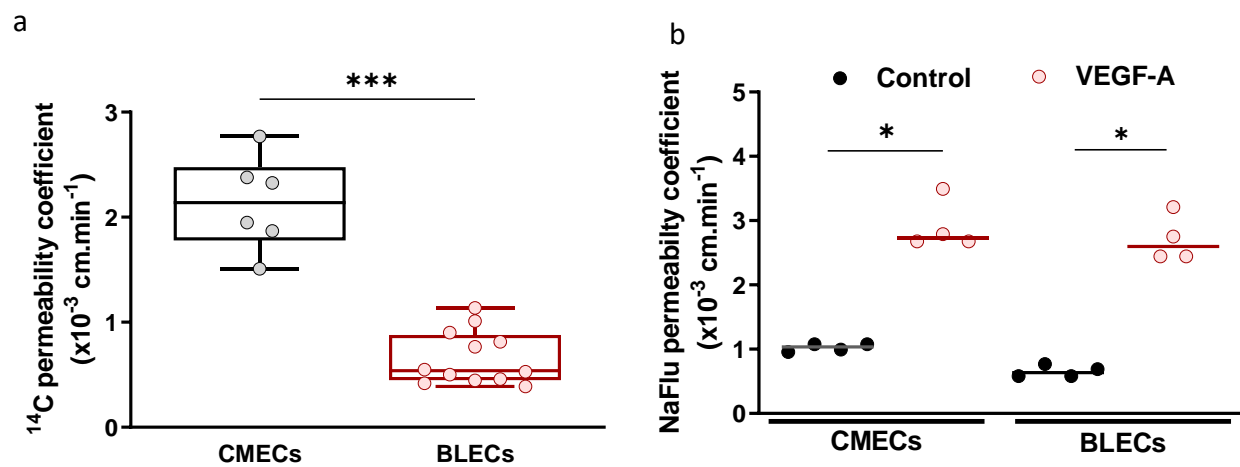

**c**

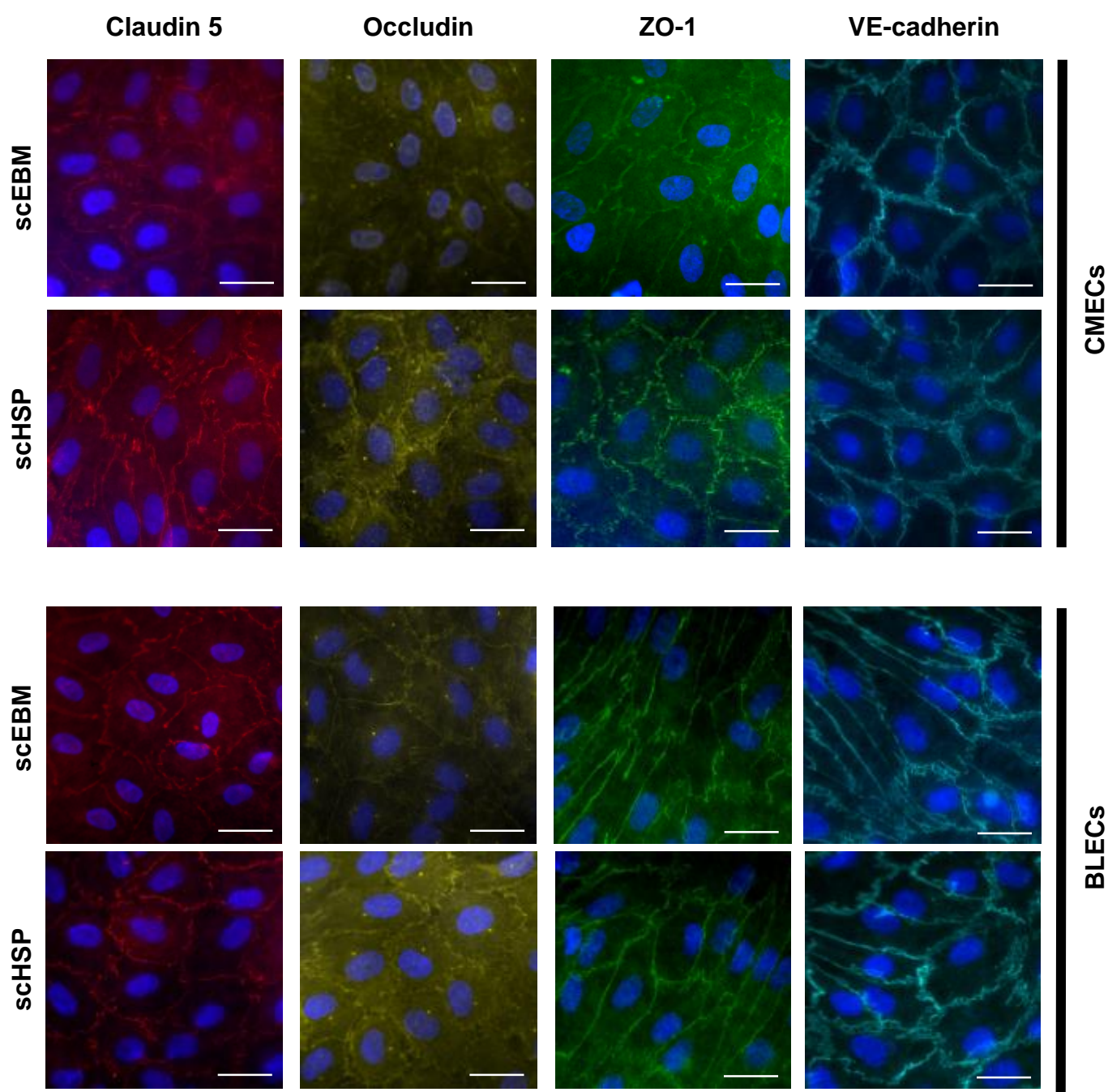

Supplement: Supplementary file 13 — Supplementary Figure 13. Effect of scHSP treatment in the endothelial barrier properties. The permeability for radiolabelled-sucrose is enhanced in CMECs compared with BLECs (a). VEGF-A (50 ng/mL) induced vascular leakage in both CMECs and BLECs (b). For (a) and (b), data represents median (with interquartile range), Wilcoxon–Mann–Whitney test, versus CMECs control. Representative images of immunofluorescence performed in CMECs (c) and BLECs (d) for detection of claudin 5, ZO-1, occludin, and VE-cadherin. Scale bar: 10 μm. [file 10020_2024_897_MOESM13_ESM.pdf]

## Slide 1
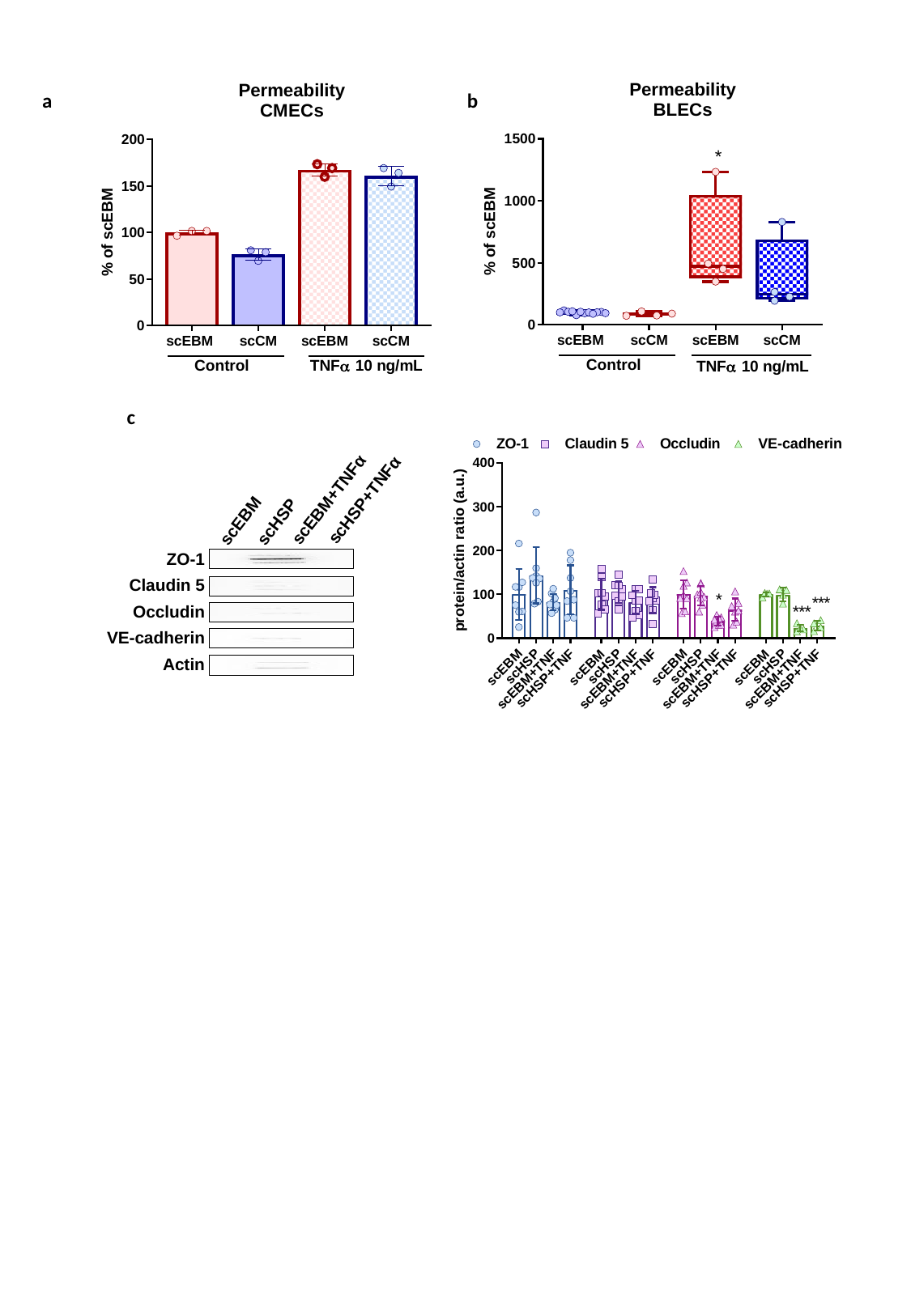

b
a
c
scHSP+TNFα
scEBM+TNFα
scEBM
scHSP
ZO-1
Claudin 5
Occludin
VE-cadherin
Actin

Supplement: Supplementary file 16 — Supplementary Figure 16. Effect of TNFα on in vitro vascular permeability. CMECs and BLECs were pre-treated (24 h) with scEBM or scHSP (5 μ/mL) and then TNFα (10 ng/mL) was administered to the cells. Whereas TNFα had no significant effect on permeability of CMECs (a), scHSP partially prevented TNFα-induced leakage in BLECs (b). WB analysis showed that scHSP partially restored the TNFα-induced downregulation of occludin in BLECs (c). Data represent median (with interquartile range, b), or mean ± SD (a, c) versus scEBM control. [file 10020_2024_897_MOESM16_ESM.pptx]
