# Supplementary material for: Secretome of brain microvascular endothelial cells promotes endothelial barrier tightness and protects against hypoxia-induced vascular leakage
Source: Mol Med. 2024 Aug 26;30:132. doi: 10.1186/s10020-024-00897-6 (PMC11348522; doi:10.1186/s10020-024-00897-6)
Supplement: Supplementary file 14 — Supplementary Figure 14. Effect of scHSP treatment in the endothelial barrier properties. Co-staining with phalloidin and ZO-1 suggests that scHSP promotes the accumulation of actin fibres at the cell borders (yellow arrowheads) and decreases its distribution across the cytoplasm of CMECs (a) and BLECs (b). scHSP up-regulates P-gp expression on BLECs, whereas it did not affect ABCA1 and BCRP expression (c). Data represent median (with interquartile range), Wilcoxon–Mann–Whitney test, versus scEBM. Scale bar: 10 μm. [file 10020_2024_897_MOESM14_ESM.pptx]

## Slide 1
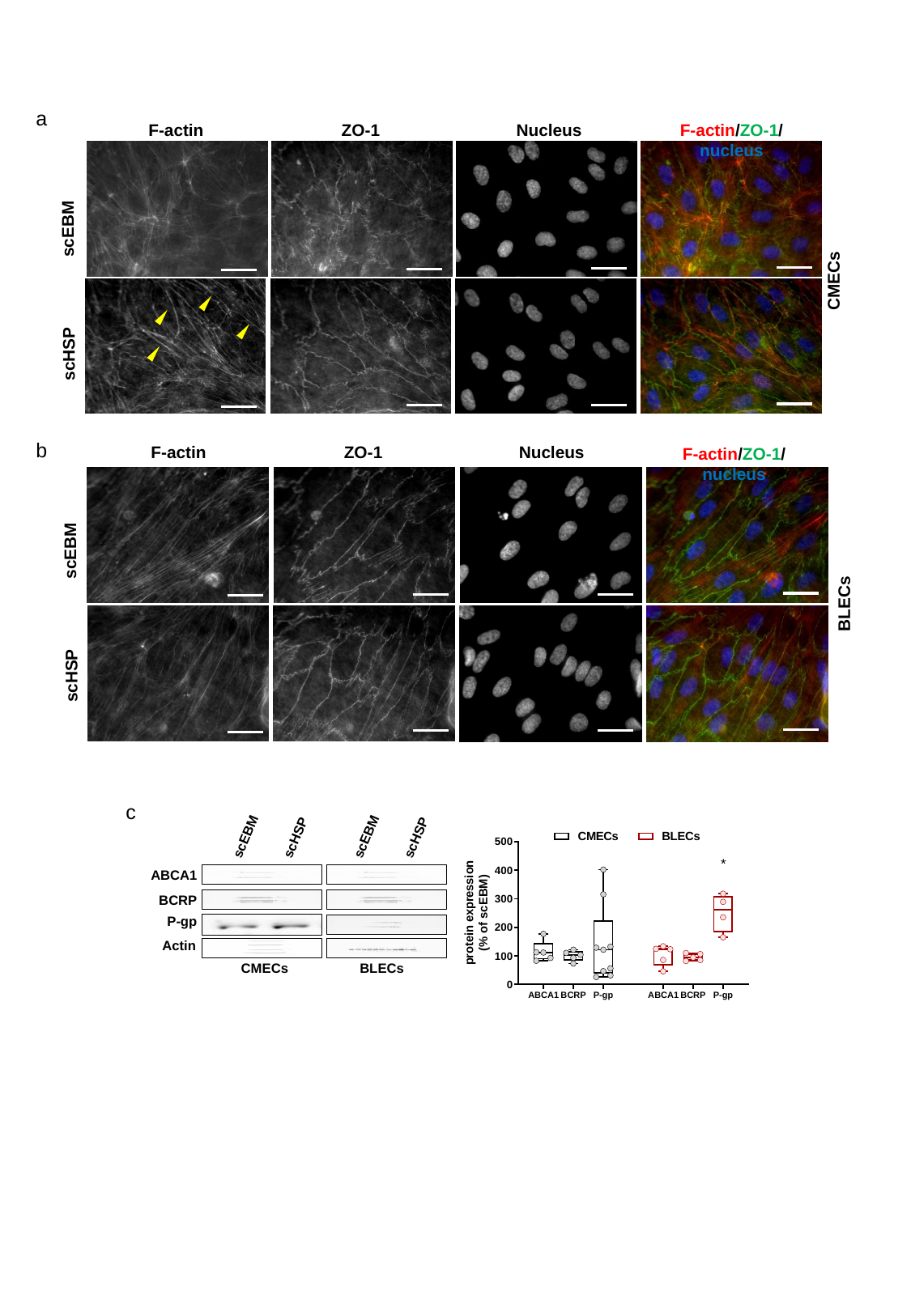

a
F-actin/ZO-1/ nucleus
Nucleus
F-actin
ZO-1
scEBM
CMECs
scHSP
b
Nucleus
F-actin
ZO-1
F-actin/ZO-1/ nucleus
scEBM
scHSP
BLECs
scEBM
scEBM
scHSP
scHSP
ABCA1
BCRP
P-gp
Actin
CMECs
BLECs
c
