# Supplementary material for: Secretome of brain microvascular endothelial cells promotes endothelial barrier tightness and protects against hypoxia-induced vascular leakage
Source: Mol Med. 2024 Aug 26;30:132. doi: 10.1186/s10020-024-00897-6 (PMC11348522; doi:10.1186/s10020-024-00897-6)
Supplement: Supplementary file 15 — Supplementary Figure 15. Effect of oxygen–glucose deprivation (OGD) on in vitro vascular permeability. OGD (6 h) had no effect on permeability of CMECs (a) while it induced vascular leakage in BLECs (b). OGD up-regulated the mRNA expression of target genes, such as HIF1A, VEGF-A, and Glut-1 (c). Western blot analysis did not show a marked effect of OGD on the expression of junctional proteins (d) however IF analysis suggested that OGD induces their accumulation in the cytoplasm (e). Data represent median (with interquartile range, b), or mean ± SD (a,c, and d), versus OGD. Scale bar: 10 μm. [file 10020_2024_897_MOESM15_ESM.pptx]

## Slide 1
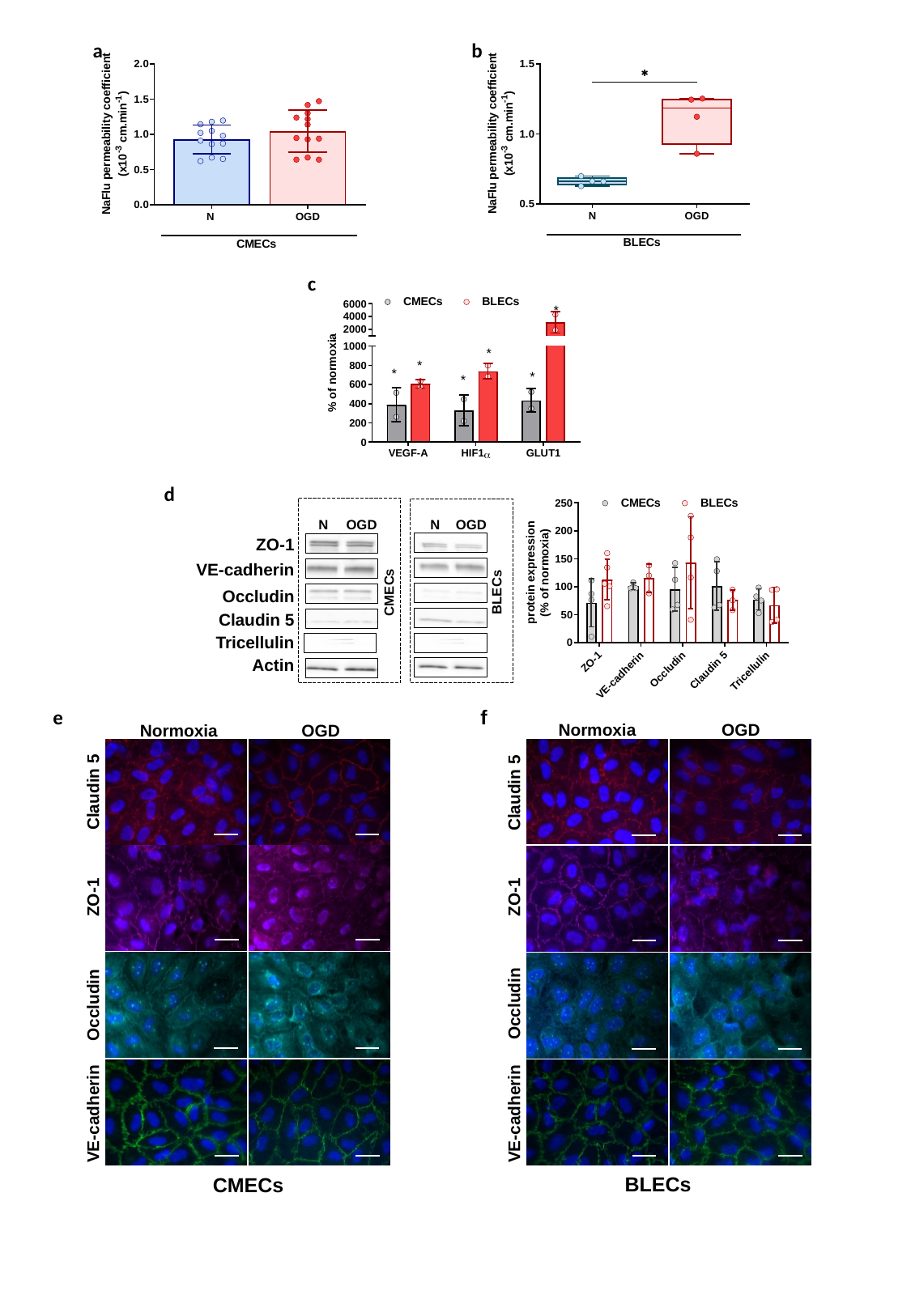

a
b
c
d
OGD
N
CMECs
N
OGD
BLECs
ZO-1
VE-cadherin
Occludin
Claudin 5
Tricellulin
Actin
f
e
Normoxia
OGD
Claudin 5
ZO-1
Occludin
VE-cadherin
BLECs
Normoxia
OGD
Claudin 5
ZO-1
Occludin
VE-cadherin
CMECs
