# Supplementary material for: Secretome of brain microvascular endothelial cells promotes endothelial barrier tightness and protects against hypoxia-induced vascular leakage
Source: Mol Med. 2024 Aug 26;30:132. doi: 10.1186/s10020-024-00897-6 (PMC11348522; doi:10.1186/s10020-024-00897-6)
Supplement: Supplementary file 18 — Supplementary Material 18. [file 10020_2024_897_MOESM18_ESM.docx]

**Supplementary Table 2:** List of genes upregulated by scHSP treatment in CMECs and their respective biological function.

| **C1R:** Complement C1r subcomponent; C1r B chain is a serine protease that combines with C1q and C1s to form C1, the first component of the classical pathway of the complement system; Belongs to the peptidase S1 family. |
| --- |
| **C1S:** Complement C1s subcomponent; C1s B chain is a serine protease that combines with C1q and C1r to form C1, the first component of the classical pathway of the complement system. C1r activates C1s so that it can, in turn, activate C2 and C4. |
| **CCL2:** C-C motif chemokine 2; Chemotactic factor that attracts monocytes and basophils but not neutrophils or eosinophils. Augments monocyte anti-tumor activity. Has been implicated in the pathogenesis of diseases characterized by monocytic infiltrates, like psoriasis, rheumatoid arthritis or atherosclerosis. May be involved in the recruitment of monocytes into the arterial wall during the disease process of atherosclerosis; Belongs to the intercrine beta (chemokine CC) family. |
| **CFB:** Complement factor B; Factor B which is part of the alternate pathway of the complement system is cleaved by factor D into 2 fragments: Ba and Bb. Bb, a serine protease, then combines with complement factor 3b to generate the C3 or C5 convertase. It has also been implicated in proliferation and differentiation of preactivated B- lymphocytes, rapid spreading of peripheral blood monocytes, stimulation of lymphocyte blastogenesis and lysis of erythrocytes. Ba inhibits the proliferation of preactivated B-lymphocytes; Belongs to the peptidase S1 family. |
| **CXCL1:** Growth-regulated alpha protein; Has chemotactic activity for neutrophils. May play a role in inflammation and exerts its effects on endothelial cells in an autocrine fashion. In vitro, the processed forms GRO- alpha(4-73), GRO-alpha(5-73) and GRO-alpha(6-73) show a 30-fold higher chemotactic activity; Chemokine ligands. |
| **CXCL2:** C-X-C motif chemokine 2; Produced by activated monocytes and neutrophils and expressed at sites of inflammation. Hematoregulatory chemokine, which, in vitro, suppresses hematopoietic progenitor cell proliferation. GRO-beta(5-73) shows a highly enhanced hematopoietic activity. |
| **DDX60:** Probable ATP-dependent RNA helicase DDX60; Positively regulates DDX58/RIG-I- and IFIH1/MDA5- dependent type I interferon and interferon inducible gene expression in response to viral infection. Binds ssRNA, dsRNA and dsDNA and can promote the binding of DDX58/RIG-I to dsRNA. Exhibits antiviral activity against hepatitis C virus and vesicular stomatitis virus (VSV); Belongs to the helicase family. |
| **HERC6:** Probable E3 ubiquitin-protein ligase HERC6; E3 ubiquitin-protein ligase which accepts ubiquitin from an E2 ubiquitin-conjugating enzyme in the form of a thioester and then directly transfers the ubiquitin to targeted substrates. |
| **HLA-B:** HLA class I histocompatibility antigen, B-7 alpha chain; Involved in the presentation of foreign antigens to the immune system; C1-set domain containing. |
| **HOXD4:** Homeobox protein Hox-D4; Sequence-specific transcription factor which is part of a developmental regulatory system that provides cells with specific positional identities on the anterior-posterior axis; HOXL subclass homeoboxes. |
| **IFI27:** Interferon alpha-inducible protein 27, mitochondrial; Promotes cell death. Mediates IFN-induced apoptosis characterized by a rapid and robust release of cytochrome C from the mitochondria and activation of BAX and caspases 2, 3, 6, 8 and 9. |
| **IFI44:** Interferon-induced protein 44; This protein aggregates to form microtubular structures; Belongs to the IFI44 family. |
| Interferon-induced protein 44-like; Exhibits a low antiviral activity against hepatitis C virus; Belongs to the IFI44 family. |
| **IFI6:** Interferon alpha inducible protein 6. |
| **IFIT1:** Interferon-induced protein with tetratricopeptide repeats 1; Interferon-induced antiviral RNA-binding protein that specifically binds single-stranded RNA bearing a 5'-triphosphate group (PPP-RNA), thereby acting as a sensor of viral single- stranded RNAs and inhibiting expression of viral messenger RNAs. Single-stranded PPP-RNAs, which lack 2'-O-methylation of the 5' cap and bear a 5'-triphosphate group instead, are specific from viruses, providing a molecular signature to distinguish between self and non-self mRNAs by the host during viral infection. |
| **IFITM1:** Interferon-induced transmembrane protein 1; IFN-induced antiviral protein which inhibits the entry of viruses to the host cell cytoplasm, permitting endocytosis, but preventing subsequent viral fusion and release of viral contents into the cytosol. Active against multiple viruses, including influenza A virus, SARS coronavirus (SARS-CoV), Marburg virus (MARV), Ebola virus (EBOV), Dengue virus (DNV), West Nile virus (WNV), human immunodeficiency virus type 1 (HIV-1) and hepatitis C virus (HCV). Can inhibit: influenza virus hemagglutinin protein- mediated viral entry, MARV and EBOV. |
| **MX1:** Interferon-induced GTP-binding protein Mx1; Interferon-induced dynamin-like GTPase with antiviral activity against a wide range of RNA viruses and some DNA viruses. Its target viruses include negative-stranded RNA viruses and HBV through binding and inactivation of their ribonucleocapsid. May also antagonize reoviridae and asfarviridae replication. Inhibits thogoto virus (THOV) replication by preventing the nuclear import of viral nucleocapsids. Inhibits La Crosse virus (LACV) replication by sequestering viral nucleoprotein in perinuclear complexes, preventing genome amplification, bud [...] |
| **NFKBIZ:** NF-kappa-B inhibitor zeta; Involved in regulation of NF-kappa-B transcription factor complexes. Inhibits NF-kappa-B activity without affecting its nuclear translocation upon stimulation. Inhibits DNA-binding of RELA and NFKB1/p50, and of the NF-kappa-B p65-p50 heterodimer and the NF-kappa-B p50-p50 homodimer. Seems also to activate NF- kappa-B-mediated transcription. In vitro, upon association with NFKB1/p50 has transcriptional activation activity and, together with NFKB1/p50 and RELA, is recruited to LCN2 promoters. Promotes transcription of LCN2 and DEFB4. |
| **OAS1:** 2'-5'-oligoadenylate synthase 1; Interferon-induced, dsRNA-activated antiviral enzyme which plays a critical role in cellular innate antiviral response. In addition, it may also play a role in other cellular processes such as apoptosis, cell growth, differentiation and gene regulation. Synthesizes higher oligomers of 2'-5'-oligoadenylates (2-5A) from ATP which then bind to the inactive monomeric form of ribonuclease L (RNase L) leading to its dimerization and subsequent activation. Activation of RNase L leads to degradation of cellular as well as viral RNA. |
| **PARP14:** Poly [ADP-ribose] polymerase 14; ADP-ribosyltransferase. By mono-ADP-ribosylating STAT1 at 'Glu-657' and 'Glu-705' and thus decreasing STAT1 phosphorylation, negatively regulates pro-inflammatory cytokines production in macrophages in response to IFNG stimulation. Mono-ADP- ribosylates STAT6 (By similarity). Enhances STAT6-dependent transcription (By similarity). In macrophages, positively regulates MRC1 expression in response to IL4 stimulation by promoting STAT6 phosphorylation. Mono-ADP- ribosylates PARP9; Poly(ADP-ribose) polymerases. |
| **PLSCR1:** Phospholipid scramblase 1; May mediate accelerated ATP-independent bidirectional transbilayer migration of phospholipids upon binding calcium ions that results in a loss of phospholipid asymmetry in the plasma membrane. May play a central role in the initiation of fibrin clot formation, in the activation of mast cells and in the recognition of apoptotic and injured cells by the reticuloendothelial system. |
| **USP18:** Ubl carboxyl-terminal hydrolase 18; Involved in the regulation of inflammatory response to interferon type 1. Can efficiently cleave only ISG15 fusions including native ISG15 conjugates linked via isopeptide bonds. Necessary to maintain a critical cellular balance of ISG15-conjugated proteins in both healthy and stressed organisms; Ubiquitin specific peptidases. |
| **XAF1:** XIAP-associated factor 1; Seems to function as a negative regulator of members of the IAP (inhibitor of apoptosis protein) family. Inhibits anti- caspase activity of BIRC4. Induces cleavage and inactivation of BIRC4 independent of caspase activation. Mediates TNF-alpha- induced apoptosis and is involved in apoptosis in trophoblast cells. May inhibit BIRC4 indirectly by activating the mitochondrial apoptosis pathway. After translocation to mitochondria, promotes translocation of BAX to mitochondria and cytochrome c release from mitochondria. |

**Legend:** Table showing description and biological function of genes upregulated in scHSP-treated CMEC. Information about each gene was obtained by using String software database. Results were obtained by performing a mRNA sequencing (TempO-Seq Analysis), as described in Methods section.
